# Cochlear Apex Triangulation Utilizing Ct Measures And Middle Ear Landmarks

**DOI:** 10.1097/ONO.0000000000000060

**Published:** 2024-08-23

**Authors:** Justin Cottrell, David Landsberger, Matt Breen, Joseph Lebowitz, Mari Hagiwara, Gul Moonis, William Shapiro, David R. Friedmann, Daniel Jethanamest, Sean McMenomey, J. Thomas Roland

**Affiliations:** 1 Department of Otolaryngology—Head and Neck Surgery, NYU Langone, New York; 2 Department of Radiology, NYU Langone, New York.

**Keywords:** Apical cochleostomy, Cochlear apex, Cochlear implant, Cochlear implant access, Cochlear implant complications, Labyrinthitis ossificans, Pitch perception

## Abstract

**Objective:**

To better characterize the cochlear apex in relation to surgically relevant landmarks to guide surgeons and improve procedural success of apical electrode placement.

**Study Design:**

Retrospective image analysis.

**Setting:**

Tertiary referral center.

**Patients:**

Cochlear implant recipients with available preoperative computed tomography (CT) imaging.

**Intervention:**

None.

**Main Outcome Measure:**

Cochlear dimensions and cochlear apex distance measures to surgically relevant middle ear landmarks and critical structures.

**Results:**

Eighty-two temporal bone CT scans were analyzed utilizing multiplanar reformats. The average lateral width of promontory bone over the cochlear apex was 1.2 mm (standard deviation [SD], 0.3). The anteroposterior distance from the round window (avg, 4.2 mm; SD, 0.5), oval window (avg, 3.3 mm; SD, 0.3), cochleariform process (avg, 2.3; SD, 0.5), and superior-inferior distance from the cochleariform process (avg, −0.9; SD, 0.8) to the cochlear apex were measured. The relationship of the cochlear apex to critical structures was highly variable.

A newly developed stapes vector was created and found to mark the posterior/superior boundary of the apex in 94% of patients. When a vector parallel to the stapes vector was drawn through the round window, it marked the anterior/inferior boundary of the cochlear apex in 89% of patients.

**Conclusions:**

This study assists in characterizing cochlear apex anatomy and its relation to surrounding structures as a means of improving procedural accuracy and reducing trauma during apical cochleostomy. Understanding both distance relationships and expected boundaries of the apex could help to inform future surgical approaches.

Stimulating the apical regions of the cochlea has previously been inaccessible to traditional precurved cochlear implant (CI) electrodes (1). A new approach has been described in which a return electrode is placed within an apical cochleostomy following standard electrode insertion, which can then reshape electrical fields to stimulate the cochlear apex (1,2). Preliminary results in patients utilizing this surgical approach and programming technique have subsequently described lower pitch perception in a majority of these subjects (1).

To achieve an accurate and less traumatic apical cochleostomy for apical electrode placement and stimulation, a thorough understanding of cochlear apex anatomy is required to gain access to the helicotrema (1). As such, a thorough understanding of cochlear apex anatomy, its relationship to surgically identifiable landmarks, and critical structures is required. While previous anatomical studies to guide surgery have focused on characterizing the round window, basal turn, and second turn of the cochlea to improve standard cochlear implant insertions, characterization of the cochlear apex is limited (3–25). This study aims to better understand cochlear apex anatomy in relation to important anatomical landmarks and critical structures to optimize procedural success, standardize technique, and facilitate preoperative planning and intraoperative decision-making.

## MATERIALS AND METHODS

The study received approval from the institutional review board (IRB# s20-01964). A single institution, retrospective radiologic anatomical review of preoperative fine-cut temporal bone computed tomography (CT) scans (<1 mm slice) of CI patients was completed. Patients were evaluated starting in January 2022, and all subsequent CI candidates were included until 41 temporal bone CT scans (82 ears: 41 left and 41 right temporal bones) were analyzed. Basic demographic information gathered for each temporal bone CT included age, sex, and laterality. Scans were excluded if there was a significant CT artifact preventing accurate measurement, middle ear disease, previous surgery, or congenital abnormalities.

Measurements were grouped into 3 categories after the number of cochlear turns was determined. The first category assessed cochlear dimensions, measuring cochlea height, apex width, and apex height. The second category looked at distances to the apex, measuring from the internal carotid artery (ICA), labyrinthine facial nerve, lateral and superior aspect of the cochlear promontory, round window, and anteroposterior/cranio-caudal (AP/CC) distances to the cochleariform process. These structures were selected as they represent critical structures to avoid, useful surgical landmarks, or areas to excavate with drilling. The third category refers to special measures. The first special measure sought to evaluate the relationship of the cochlear apex to a line parallel to the anterior wall of the internal carotid. The second 2 special measures utilized a vector line through the anterior and posterior crus of the stapes, to evaluate its utility in triangulating superior and inferior boundaries of the apex.

**FIG. 1.**
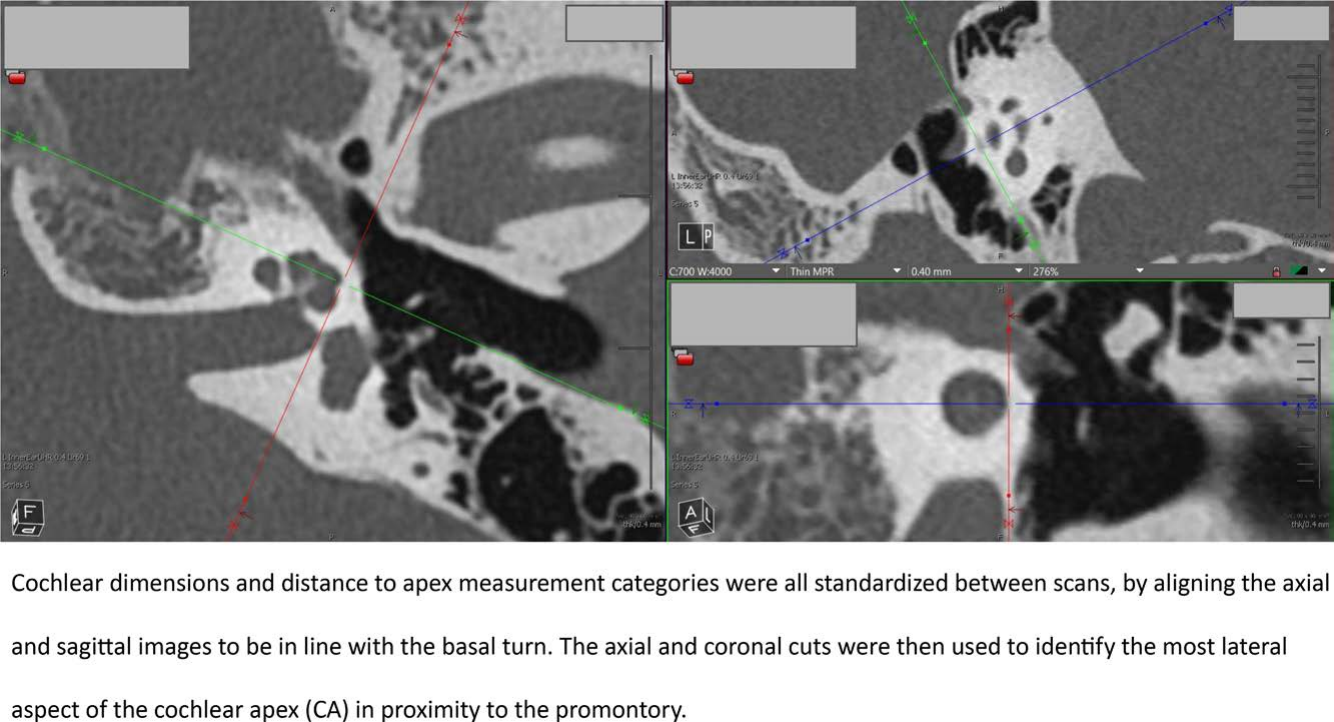
Image standardization. Cochlear dimensions and distance to apex measurement categories were all standardized between scans, by aligning the axial and sagittal images to be in line with the basal turn. The axial and coronal cuts were then used to identify the most lateral aspect of the cochlear apex (CA) in proximity to the promontory.

**FIG. 2.**
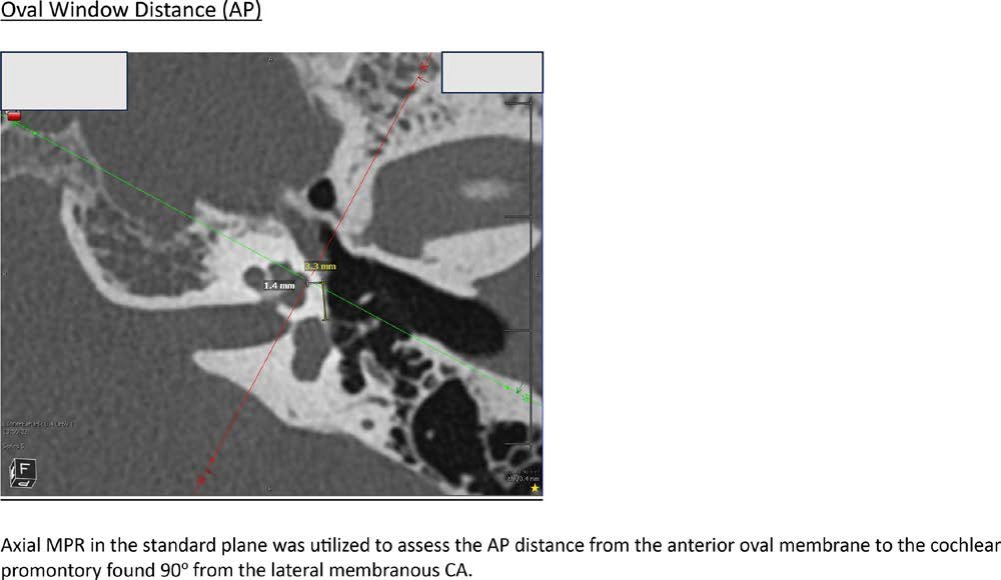
Measuring the oval window distance (anteroposterior [AP]). Axial MPR in the standard plane was utilized to assess the AP distance from the anterior oval membrane to the cochlear promontory found 90^o^ from the lateral membranous cochlear apex (CA).

A full list of measurements, and measurement procedures, are included in the corresponding article Supplemental Figure; http://links.lww.com/ONO/A32. All measures were carried out in Visage Imaging v7.1.18. The measurement protocol was developed through the collaboration of the neurotology and neuroradiology group at the study center. A Fellow of the Royal College of Surgeons of Canada in otolaryngology—head and neck surgery completed measurements. Cochlear dimension and distance to apex measurement categories were all standardized between scans, by aligning the axial and coronal images to be in line with the basal turn. The axial and sagittal cuts were then used to identify the most lateral aspect of the apex in proximity to the promontory (Fig. 1). This also represented the midportion of the lateral aspect of the apical turn in terms of height, as this is where the lateral aspect of the apical turn is the widest. This was deemed to be the most surgically relevant portion of the apex to complete measurements from, as it is in closest proximity to the middle ear space, and likely to be the first portion of the apex encountered when drilling. Figure 2 provides an example of how the distance measures were completed from the oval window to the cochlear apex. Measurements from the round window occurred from the anterior round window membrane so that measures would remain applicable once the surgeon removes the niche.

**FIG. 3.**
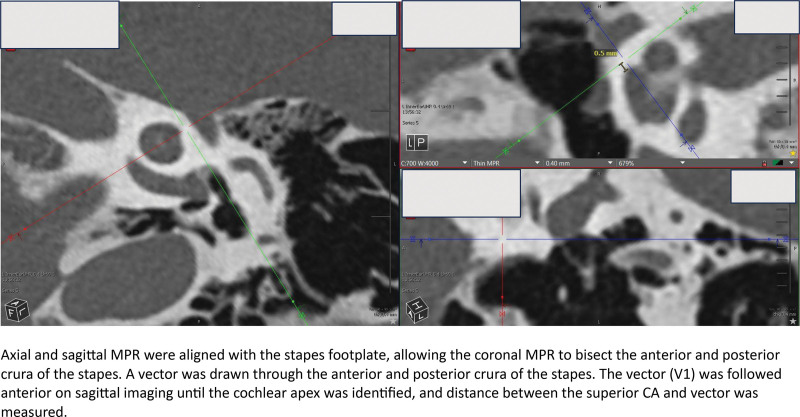
Stapes vector. Axial and sagittal MPR were aligned with the stapes footplate, allowing the coronal MPR to bisect the anterior and posterior crura of the stapes. A vector was drawn through the anterior and posterior crura of the stapes. The vector (V1) was followed anterior on sagittal imaging until the cochlear apex was identified, and the distance between the superior cochlear apex (CA) and vector was measured.

The majority of the measurements to the cochlear apex utilized the most lateral aspect of the apex as a reference point, which is distal to the second turn of the cochlea. For measurements evaluating apical width and closest proximity (ex. ICA and labyrinthine facial nerve), the most apical 360^o^ was utilized, which incorporates portions of the second turn but is deemed important to characterize when assessing width or critical structure relationships (Supplemental Figure; http://links.lww.com/ONO/A32). The category of special measures required additional multiplanar reformation adjustments, which are defined in the Supplemental Figure; http://links.lww.com/ONO/A32. Figure 3 provides an example of how one of the special measures, the stapes vector (V1), was evaluated. Cadaveric dissection was completed on a temporal bone that had not been imaged to correlate with the findings from image analysis and to provide a visual representation of imaging measures.

## RESULTS

Eighty-two temporal bones (each side of 41 patient scans, representing 50:50 left and right temporal bone representation) were analyzed in 44 (54%) male bones and 38 (46%) female bones, with an average age of 56.4 (standard deviation [SD], 26.8; range: 0.2–92.5yo) (Table 1). The majority of temporal bones demonstrated 2.5 turns (62; 76%), with 16 bones (20%) demonstrating 2.75 turns, 3 bones (4%) demonstrating 2.25 turns, and 1 bone (1%) demonstrating 3 turns (Table 1). The results of all measurements are presented in Tables 1 and 2.

**TABLE 1.** Temporal bone details

		(n)
Total ears	Total	82
Age		(Years)
	Mean (Range)	56.4 (0.2–92.5)
	SD	26.8
Sex		% (n)
	Male	54 (44)
	Female	46 (38)
Ear laterality		
	Left	50 (41)
	Right	50 (41)
Cochlear turns		
	2.25	4 (3)
	2.5	76 (62)
	2.75	20 (16)
	3	1 (1)

**TABLE 2.** Cochlear apex measurements

	Measurement (mm)^a^	Min	Max	Mean	Standard deviation
Cochlea dimensions	Cochlea height	2.7	4.0	3.4	0.3
	Apex width	2.7	4.0	3.3	0.3
	Apex height	0.7	1.7	1.0	0.2
Distance to apex	Internal carotid^b^	1.4	8.9^b^	3.0	1.2
	Labyrinthine facial nerve	0.6	2.3	1.4	0.4
	Lateral aspect of cochlear promontory	0.6	2.2	1.2	0.3
	Superior aspect of cochlear promontory	0.8	2.1	1.3	0.3
	Round window (AP distance)	3.1	5.4	4.2	0.5
	Oval window (AP distance)	2.4	3.9	3.3	0.3
	Cochleariform process (AP distance)	1.0	3.8	2.3	0.5
	Cochleariform process (height)	−2.6	1.0	−0.9	0.8
	Tensor tympani level	−1.6	1.3	0.2	0.6
Special measures	Anterior carotid distance	−5.3	4.4	1.2	1.8
	Stapes vector (V1)	−2.4	0.8	−0.5	0.5
	Stapes vector (V2)	−1.6	1.7	0.4	0.6

a Refer to supplemental for measurement definitions.

b 6 bones did not have ICA visible on coronal imaging at level of CA.

AP indicates anteroposterior; CC, cranio-caudal.

The mean height of the cochlea was 3.4 mm (SD, 0.3), the height of the apex 1.0 mm (SD, 0.2 mm), and the width of the apex 3.3 mm (SD, 0.3). The range between maximum and minimum values for cochlea dimension measures was <1.3 mm. The SD for the distance to apex measure category was similar when evaluating the bone surrounding the apex or structures within the otic capsule, with a mean lateral promontory width of 1.2 mm (SD, 0.3), superior promontory width of 1.3 mm (SD, 0.3), and distance to labyrinthine facial 1.4 (SD, 0.4). Distance to the ICA which utilized the shortest distance from apex to the carotid utilizing the coronal slice at the level of the most lateral aspect of the cochlear apex (see Supplemental Figure; http://links.lww.com/ONO/A32), was highly variable, with a mean of 3 mm (SD, 1.2), and range of 1.4–8.9 mm for patients with feasible measures. Six bones (7.3%) did not have the ICA visible in the standardized image cut analyzed at the level of the cochlear apex. The minimum distance to critical structures such as the ICA and labyrinthine portion of the facial nerve was 1.4 mm and 0.6 mm, respectively. The mean round window AP distance was 4.2 mm (SD, 0.5), oval window AP distance was 3.3 mm (SD, 0.3), AP cochleariform distance was 2.3 mm (SD, 0.5), and cochleariform height distance demonstrating more variability of −0.9 mm (SD, 0.8). Although most temporal bones demonstrated a lateral border of the cochlear apex that was inferior to the cochleariform process, there were 5 (6.1%) patients where the lateral border of the cochlear apex was superior (max 1 mm). The height of the tensor tympani at the lateral aspect of the cochlear apex was variable (Table 2).

**FIG. 4.**
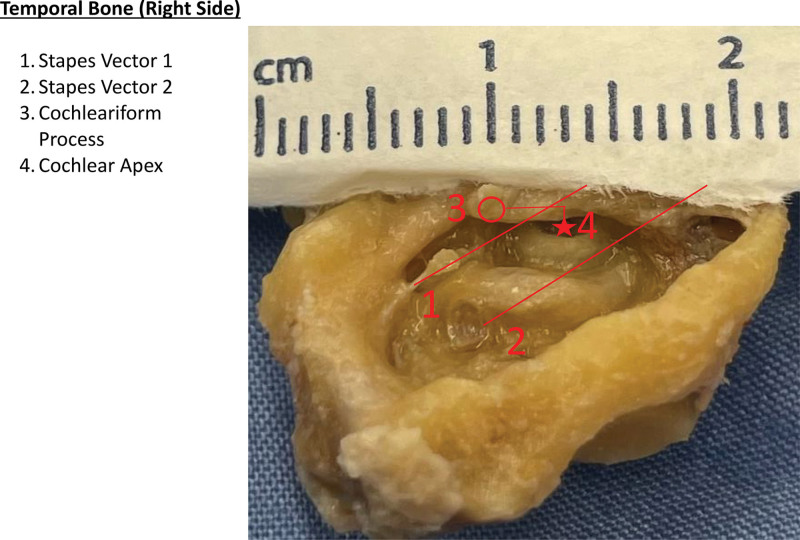
Cadaveric dissection. Temporal bone (right side) showing (1) stapes vector 1, (2) stapes vector 2, (3) cochleariform process, (4) cochlear apex.

Looking at special measures, the relation of the anterior ICA plane was highly variable, with a mean of 1.2 mm (SD, 1.8). Utilizing the stapes vector, the average distance from V1 was −0.5 mm (SD, 0.5 mm), and V2 0.4 mm (SD, 0.6 mm). This translated to V1 acting as a posterior/superior boundary for the CA in 94% of patients, and V2 acting as an anterior/inferior boundary for the CA in 89% of patients. Cadaveric temporal bone assessment of the stapes vectors was in keeping with CT measures (Fig. 4).

## DISCUSSION

Preliminary research has demonstrated the potential benefit of lower pitch perception with the additional placement of a single electrode within an apical cochleostomy together with a standard array placed with the usual approach in the nonmalformed cochlea. Accurate placement of the electrode is paramount, however, and at this time it has only been studied at a high-volume cochlear implant center (1,2). In-vivo studies have shown current flow is minimally impacted when the implant ground electrode is placed in variable extracochlear locations, and cadaveric studies show extracochlear apical placement results in insufficient intra-cochlear apical stimulation (2,26). Previous indications for apical cochleostomies were rare, often reserved for severe labyrinthine ossification of the basal and middle turns, or as a result of misplaced middle-turn cochleostomy (3,4). The experience of surgeons performing apical cochleostomy is therefore variable, and with the apex lacking a visualized entry point into the promontory, procedural uptake and quality may be hindered without a well-described approach. This study provides a newly described mechanism for determining cochlear apex boundaries utilizing identifiable middle ear landmarks, in addition to distances and relationships that can guide surgeons to achieve reliable apical electrode placement in both the nonmalformed and ossified cochlea.

Historically, the position of the cochlear apex is described to be medial to the tensor tympani muscle and anterior to the cochleariform process and oval window (1). Senn *et al.* (4) evaluated imaging of 8 patients and 3 cadaveric temporal bones that attempted retrograde array insertions near the cochlear apex using a cochleostomy 2.5 mm anterior to the oval window and 1 mm inferior to the cochleariform process. Reliable placement of the array into the apex was not achieved in cadaveric specimens using surface landmarks, and 62.5% (5/8) of implanted ears demonstrated middle-turn insertions (4). Isaacson *et al.* (3) sought to perform a middle-turn cochleostomy in 10 cadaveric temporal bones by drilling 2 mm anterior to the oval window and just inferior to the cochleariform process (3). This resulted in 80% (8/10) of cochleostomies occurring at the basal and middle-turn transition, 10% (1/10) at the scala vestibuli of the proximal basal turn, and 10% (1/10) at the cochlear apex (3). While such placement variability is reasonable within the setting of labyrinthitis ossificans, it is not acceptable for the purposes of an apical cochleostomy during standard cochlear implantation where minimal trauma is desired, and therefore a better understanding of landmarks is needed.

A 1 mm cochleostomy is required in the apex to facilitate the placement of currently available ground electrodes. The average width of promontory bone that must be removed to reach the entry point to the apex is on average of 1.2 mm (range, 0.3–2.2 mm). Based on measures completed in this study, the variability of apex location can be appreciated (Table 2). We start with assessing the optimal distance anteriorly to initiate drilling. The AP distance from the oval window, round window, and cochleariform process to the lateral aspect of the cochlear apex ranges between 2.4–3.9 mm, 3.1–5.4 mm, and 1.0–3.8 mm, respectively. With an average AP distance from the oval window of 3.3 mm, it becomes evident why historical teaching to use 2.5 mm resulted in high levels of middle-turn insertions (4). Different landmarks provide variable clinical utility, with the oval window having the smallest SD at 0.3 mm, compared with the round window (SD, 0.5 mm) and cochleariform process (SD, 0.5 mm). On CT scan, the most anterior border of the oval window was utilized for measurements, however this may be difficult to appreciate intraoperatively depending on the view and level of bone overhang. The round window provides some benefits in this regard, in that the niche is frequently drilled flush with the round window in surgery, and can provide a reliable starting landmark. While the average AP distance from the cochleiform process was 2.3 mm, the 90th percentile was 2.9 mm, and 99th percentile was 3.4 mm. With a desire to avoid the middle-turn entry, one may consider starting more distal from the average AP measures and working posteriorly to avoid inaccurate entry into the basal regions of the cochlea.

The next step is approximating the cochlear apex height. We utilized the inferior border of the cochleariform process as our primary landmark for determining height and found an average lateral apical height to be 0.9 mm below the cochleariform process (SD, 0.8 mm). There were 5 patients (6.1%) that had their most lateral aspect of the cochlear apex located superior to the inferior border of the cochleariform process. At the 90th percentile of temporal bones, the lateral aspect of the cochlear apex was at the level of the inferior cochleariform border, and at the 99th percentile, the range extended to 0.7 mm above the inferior border of the cochleariform process. When evaluating the extent of inferior variability, the height of the 5th percentile of temporal bones was 2.2 mm below the cochleariform process, and the height of the 1st percentile was 2.4 mm below. It appears based on this data that starting the cochleostomy at a height of 0.9 mm below the cochleariform process is a reasonable approximation, and is a location of safety from the facial nerve. Due to the variability in the superior/inferior trajectory of the tensor tympani muscle along the eustachian tube from the cochleariform process, assessment of the relationship on preoperative CT scan, when available, may assist in knowing whether drilling underneath the muscle belly is expected.

The range seen for each measure highlights how additional exposure may be required after drilling initiation. We sought to design reliable “gates” that can be used to facilitate a wider front for safety and accuracy. A novel reference we have developed to assist with this is 2 stapes vectors. The first vector (V1) is formed by drawing a line through the anterior and posterior crus of the stapes, providing a reliable threshold to mark the posterior/superior boundary of the apex in 94% of patients. Translating a parallel vector to V1, at the level of the anterolateral aspect of the round window provides the second vector (v2), providing a reliable threshold to mark the anterior/inferior boundary of the apex in 89% of patients. Both gates should still be kept in context with general safety landmarks, recognizing the location of the facial nerve superior and medial to the cochleariform process.

Looking at the proximity of critical structures, we noted the internal carotid artery and labyrinthine facial nerve were at an average distance of 3 mm for patients inferiorly, and 1.4 mm superiorly in the coronal plane, respectively. As every effort should be made to avoid these structures, respecting the minimum found distance of 1.4 mm to the internal carotid artery inferiorly, and 0.6 mm to the labyrinthine facial nerve superiorly in the coronal plane is advised. Previous cadaveric studies show the average distance from the carotid artery to the round window to be 7.9 mm (5.3–11.0 mm), and the cochleariform process 9.3 mm (8–11mm) (13,27). Although the carotid must still be considered with apical drilling, the proximity from the apex is further than what is seen with the basal turn, in which the cochlear-carotid interval can be as close as 0.2 mm, separated only by the otic capsule (10–12,15). Previous literature has described the relationship of the basal turn to the posterior border of the petrous carotid (28). In this research, a similar special measure was created using the anterior border of the carotid apical turn. This measurement system yielded seemingly poor utility due to the wide range seen (−5.3 to 4.4 mm). A quick assessment of the CT scan if available to determine whether the carotid is located lateral to the apex could provide reassurance for a lower risk of injury.

These measures have been completed by manipulating MPR images to facilitate standardization in the plane with the cochlea. As such, the translation of these measurements and relationships to surgical procedures could offer different levels of utility depending on the approach and techniques employed. Different angulations of the cochlea, for example, which have been shown to vary between 30.8^o^–70.3^o^ in transverse plane, and 1–50.5^o^ in the sagittal plane, may impact the height of structures from each other when a patient is supine and visualized under the microscope (29). To help mitigate these forms of error, measurement strategies were employed to increase the pragmatic nature of the findings. For example, the AP distances were measured along the promontory bone, rather than a true anterior and posterior direction, so that they could be followed easily when looking at the bone during surgery.

Skill and experience within the temporal bone are crucial to good outcomes and employing new techniques. The findings in this study may assist in the development of safe and consistent apical cochleostomy for surgeons, and new apical electrode designs, as the benefits of apical insertion are realized. Additional anatomic outliers may exist, and therefore careful CT evaluation when available is advised. Furthermore, as this study utilizes image analysis of submillimeter distance, human error may arise when selecting anatomical reference points and completing subsequent measurements. Our cadaveric temporal bone dissection gives early validation of our results; however, it was completed following full resection of the temporal bone and wide exposure, which does not reflect the experience and visualization that would be encountered during a transmastoid approach. Moving forward, research on cadaveric specimens in the surgical position will be helpful to elucidate real-life applicability of these measures in improving apical cochleostomy placement.

## CONCLUSIONS

This study assists in characterizing cochlear apex anatomy and its relation to surrounding structures. A more accurate blueprint for performing an apical cochleostomy if indicated can improve future work to refine surgical steps, technique, and technology, to improve safety and outcomes.

## FUNDING SOURCES

This research is funded through an NIH R21 grant (DC019743). Principle investigators include Dr. David Landsberger and Dr. J. Thomas Roland Jr.

## CONFLICTS OF INTEREST STATEMENT

Dr. J Thomas Roland Jr. is a consultant for Cochlear Americas and received research funding for CI-related projects that are not affiliated with this research. Other authors disclose no conflicts of interest.

## DATA AVAILABILITY STATEMENT

The datasets generated during and/or analyzed during the current study are not publicly available but are available from the corresponding author on reasonable request.

## Supplementary Material


