# Supplementary material for: Cochlear Apex Triangulation Utilizing Ct Measures And Middle Ear Landmarks
Source: Otol Neurotol Open. 2024 Aug 23;4(3):e060. doi: 10.1097/ONO.0000000000000060 (PMC11424059; doi:10.1097/ONO.0000000000000060)

Supplemental: Measurement Methodology

Cochlear dimensions and distance to apex measurement categories were all standardized between scans, by aligning the axial and sagittal images to be in line with the basal turn.

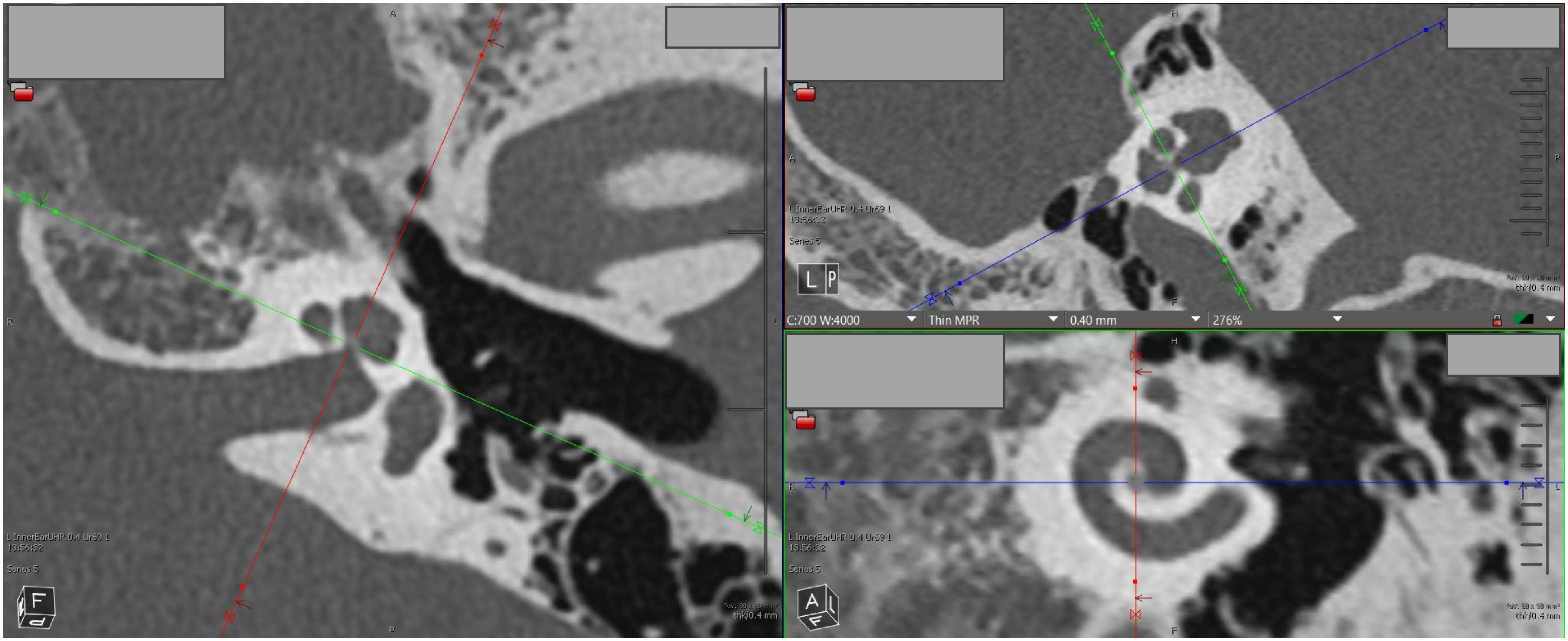

The axial and coronal cuts were then used to identify the most lateral aspect of the cochlear apex (CA) in proximity to the promontory.

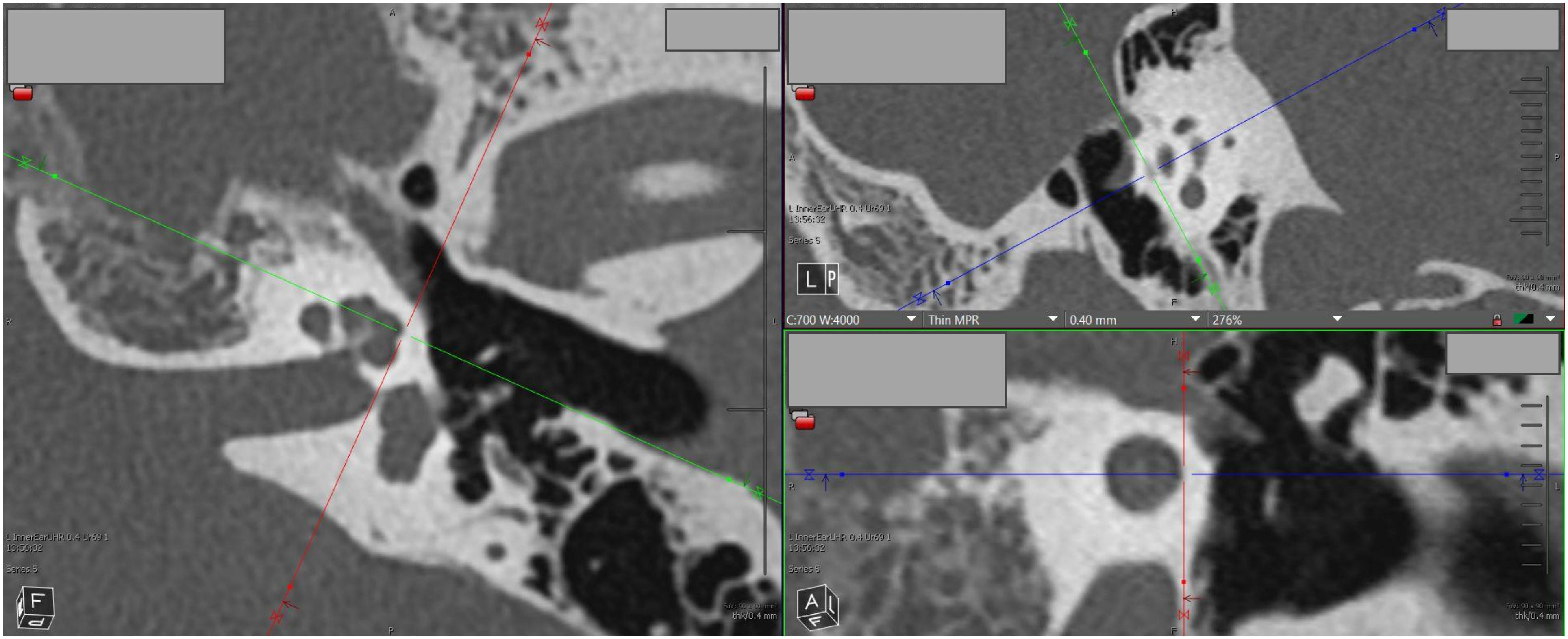

Positive numbering was provided to measurements in which the structure was anterior or lateral to the CA, and a negative number was assigned to structures placed posterior or medial.

Cochlear Turns

Coronal MPR in the standard plane was utilized to assess the number of cochlear turns in ¼ turn increments.

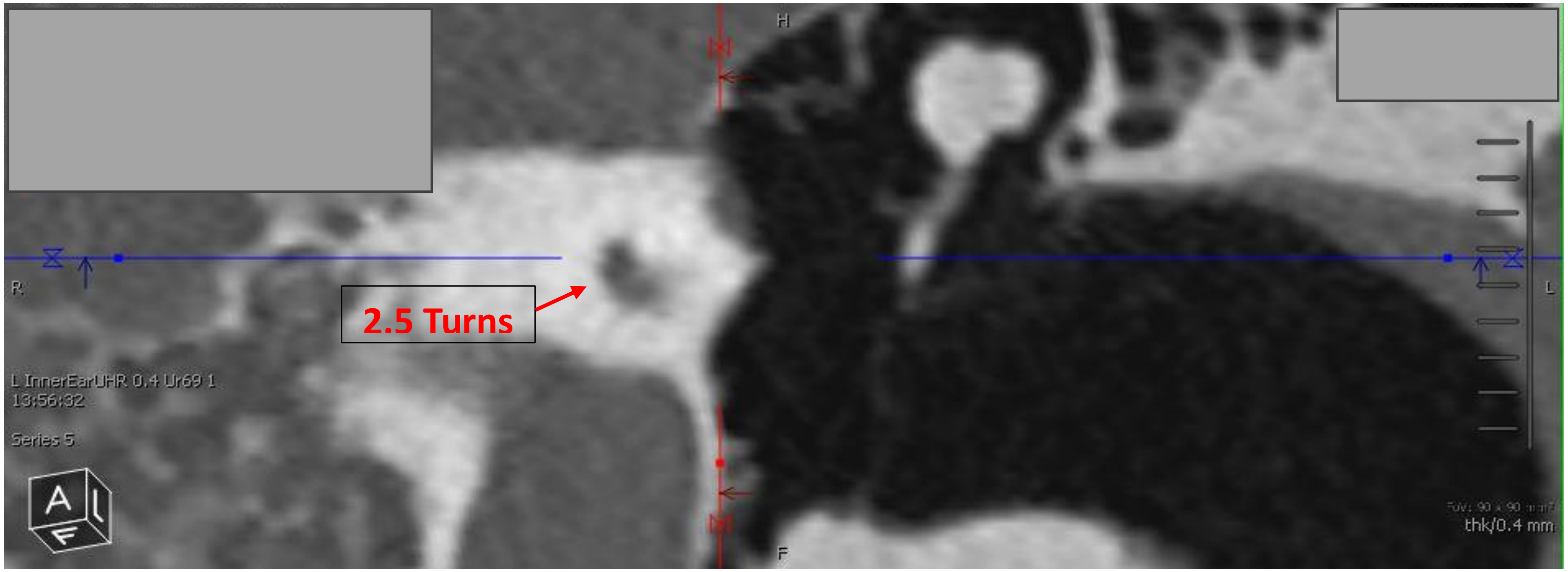

Cochlear Dimensions

Cochlear Height

The height of the entire cochlea was measured from the superior aspect of the CA to the cochlear base in sagittal plane

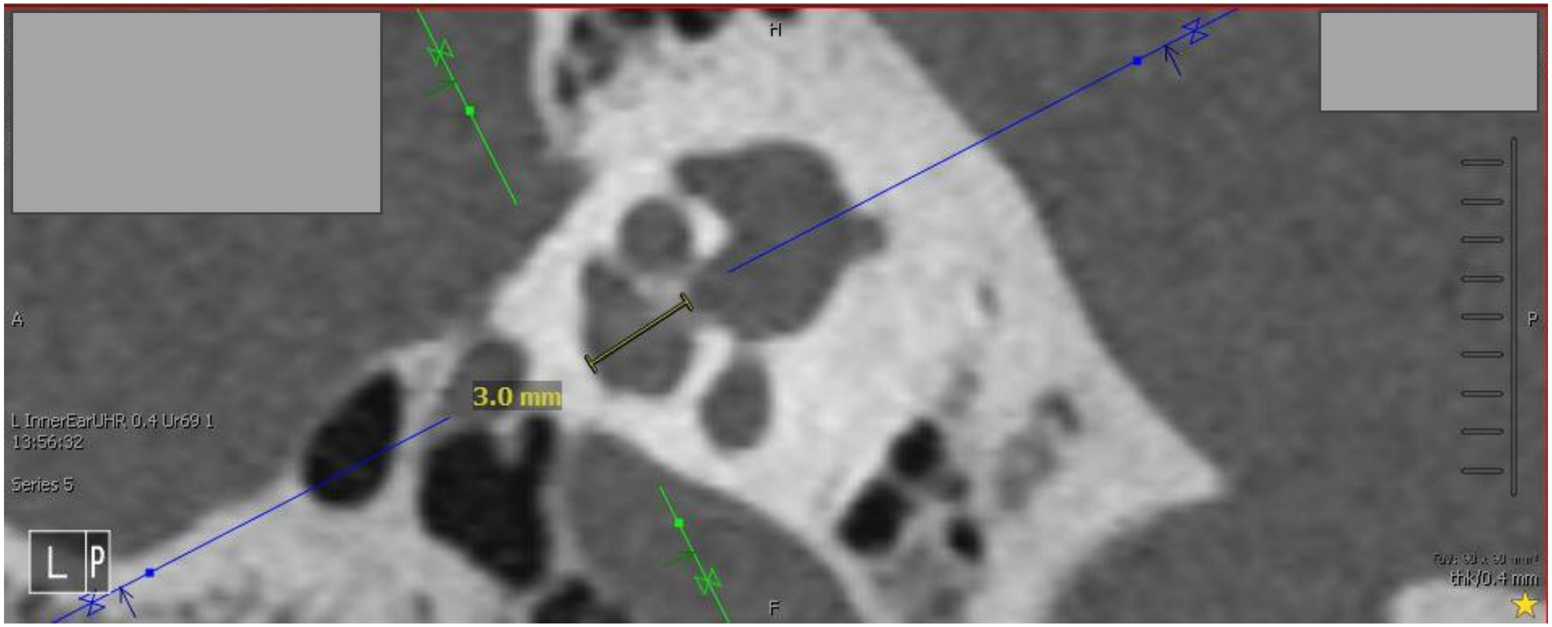

Apex Width

Coronal MPR in the standard plane was utilized to measure the width of the most apical 360° of the cochlear apex.

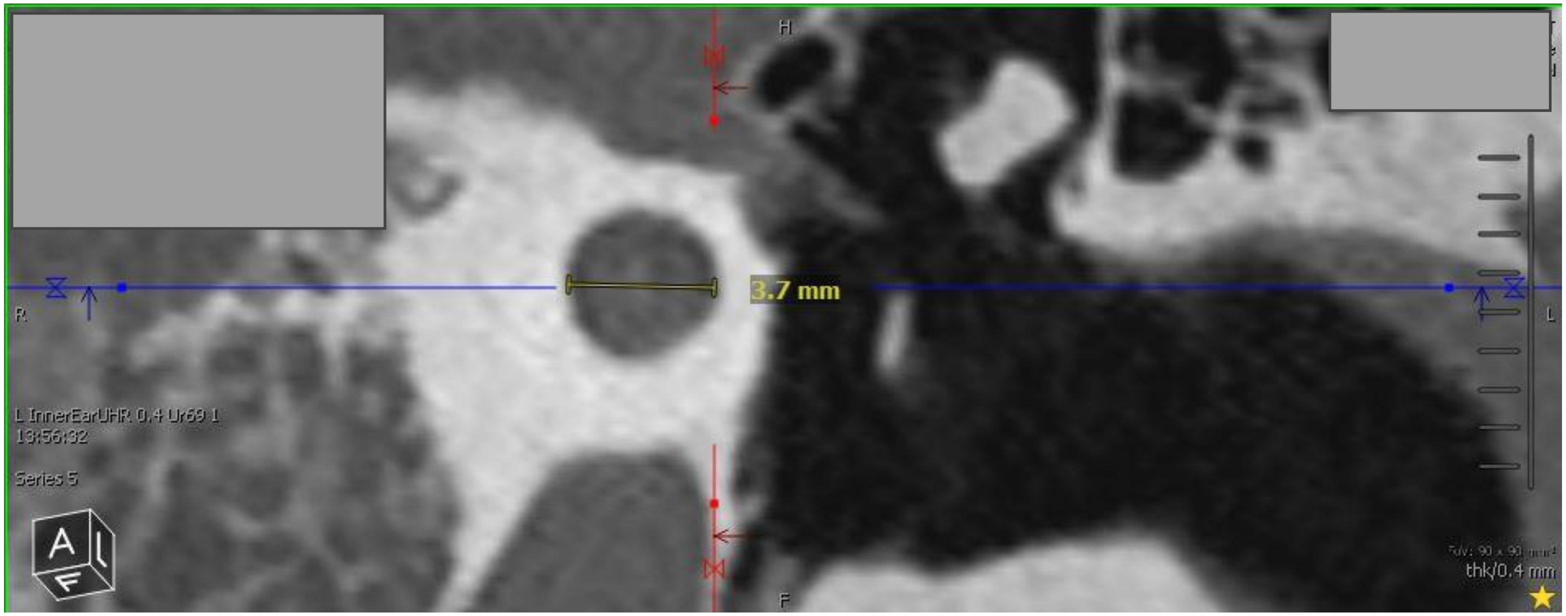

<u>Apex Height</u>

Axial MPR in the standard plane was utilized to measure the height of the cochlear apex at its lateral turn.

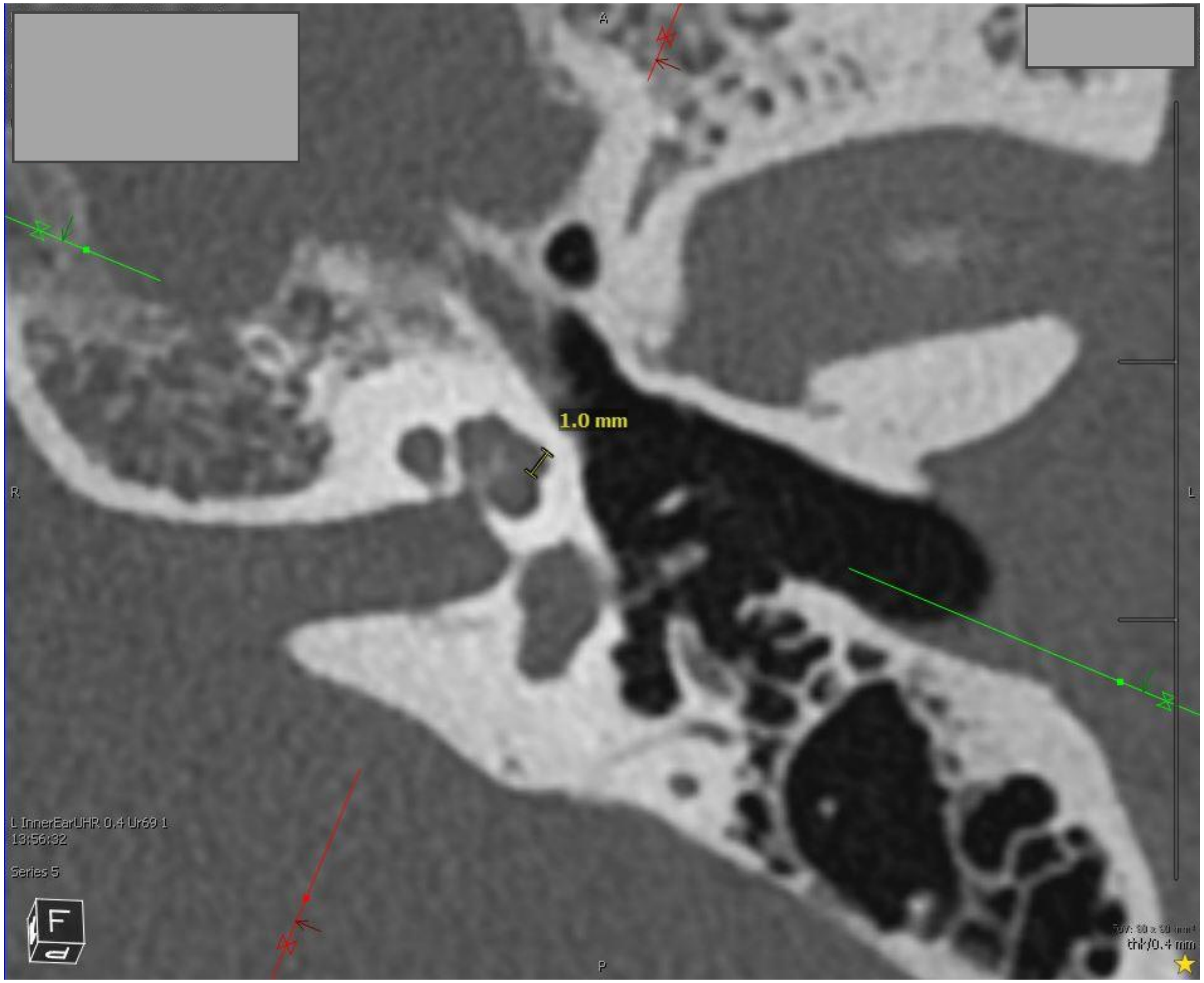

Distance to the Apex

Lateral Promontory Width

Axial MPR in the standard plane was utilized to measure the width of promontory bone, between the lateral aspect of the membranous CA, to the point where it intersects the bony promontory at a 90° angle.

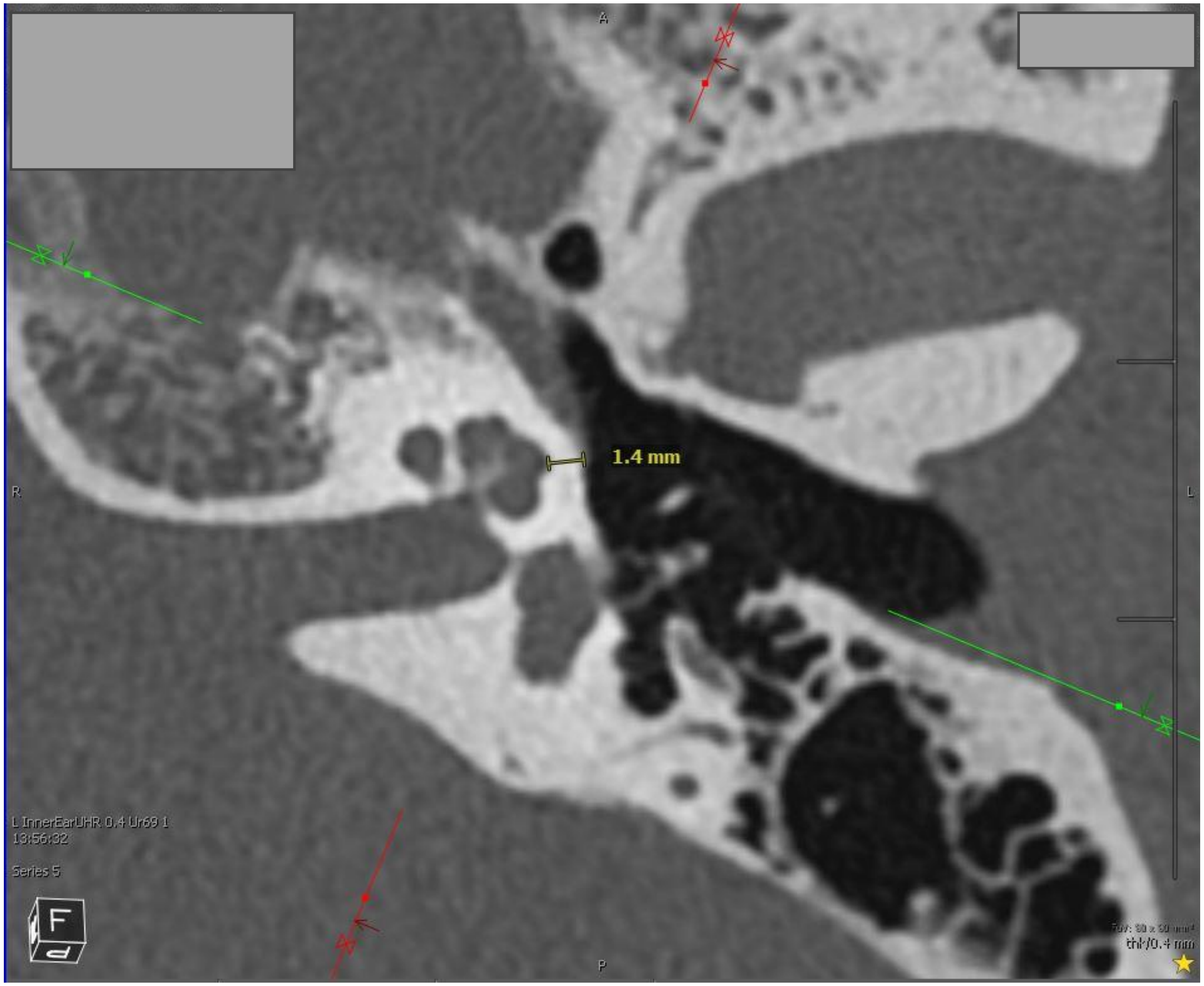

<u>Superior Aspect of Cochlear Promontory</u>

Sagittal MPR in the standard plane was utilized to assess the distance between the lateral aspect of the membranous CA, and the bony promontory found vertically.

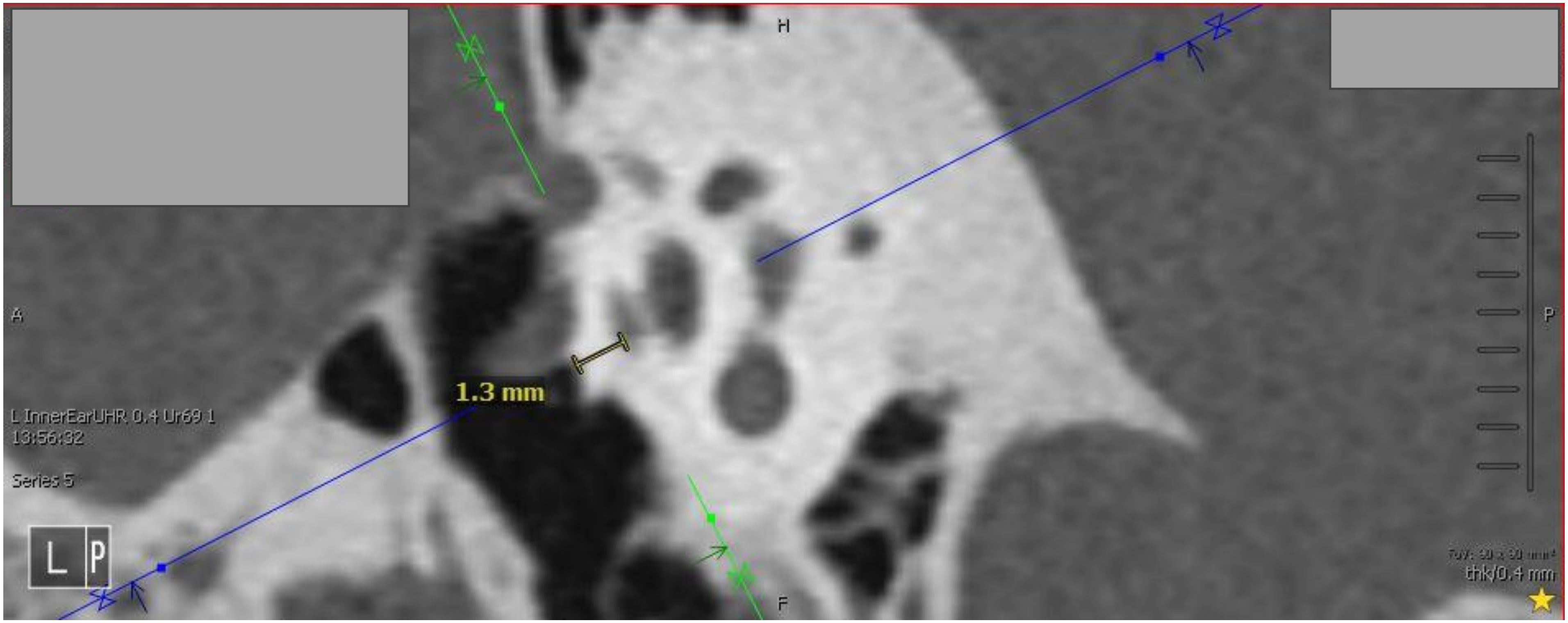

Round Window Distance (AP)

Axial MPR in the standard plane was utilized to assess the AP distance from the anterior round window membrane to the cochlear promontory found 90º from the lateral membranous CA.

Step 1: Identify anterior border of round window membrane and initiate AP measurement

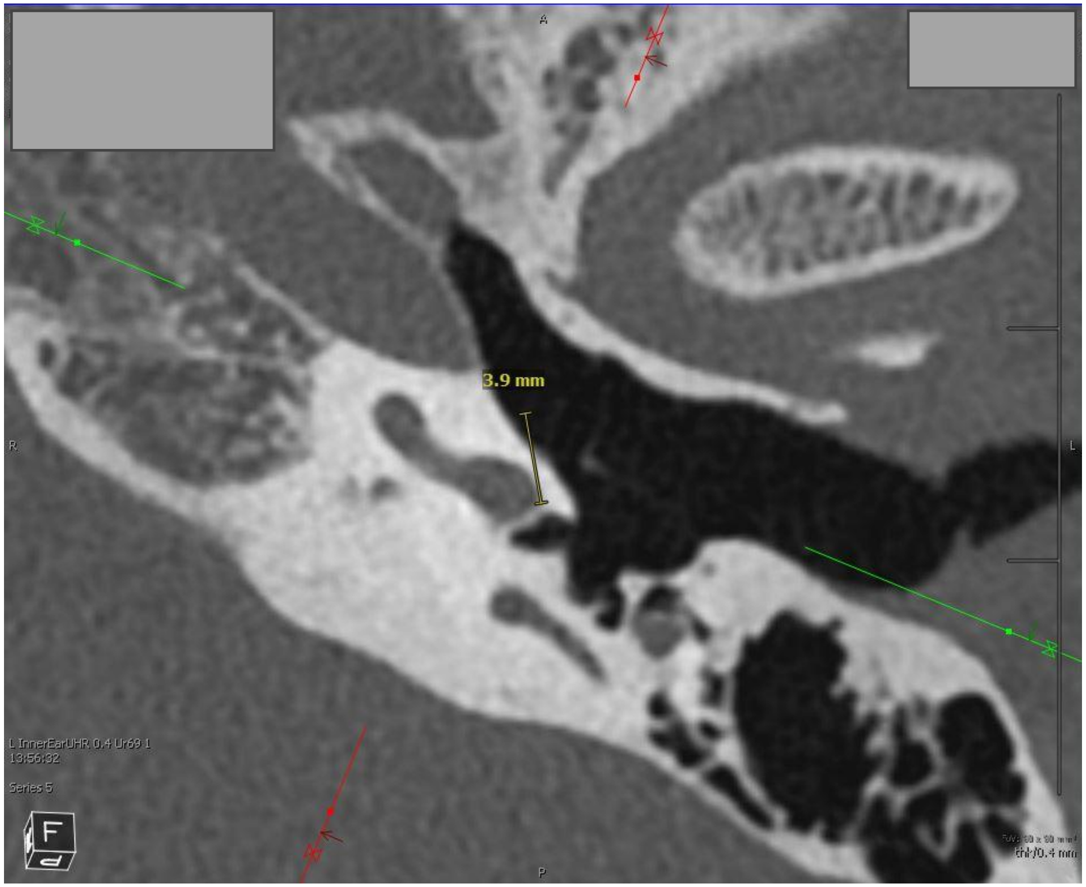

Step 2: Scroll through axial images until reach level of lateral apex to complete measurement

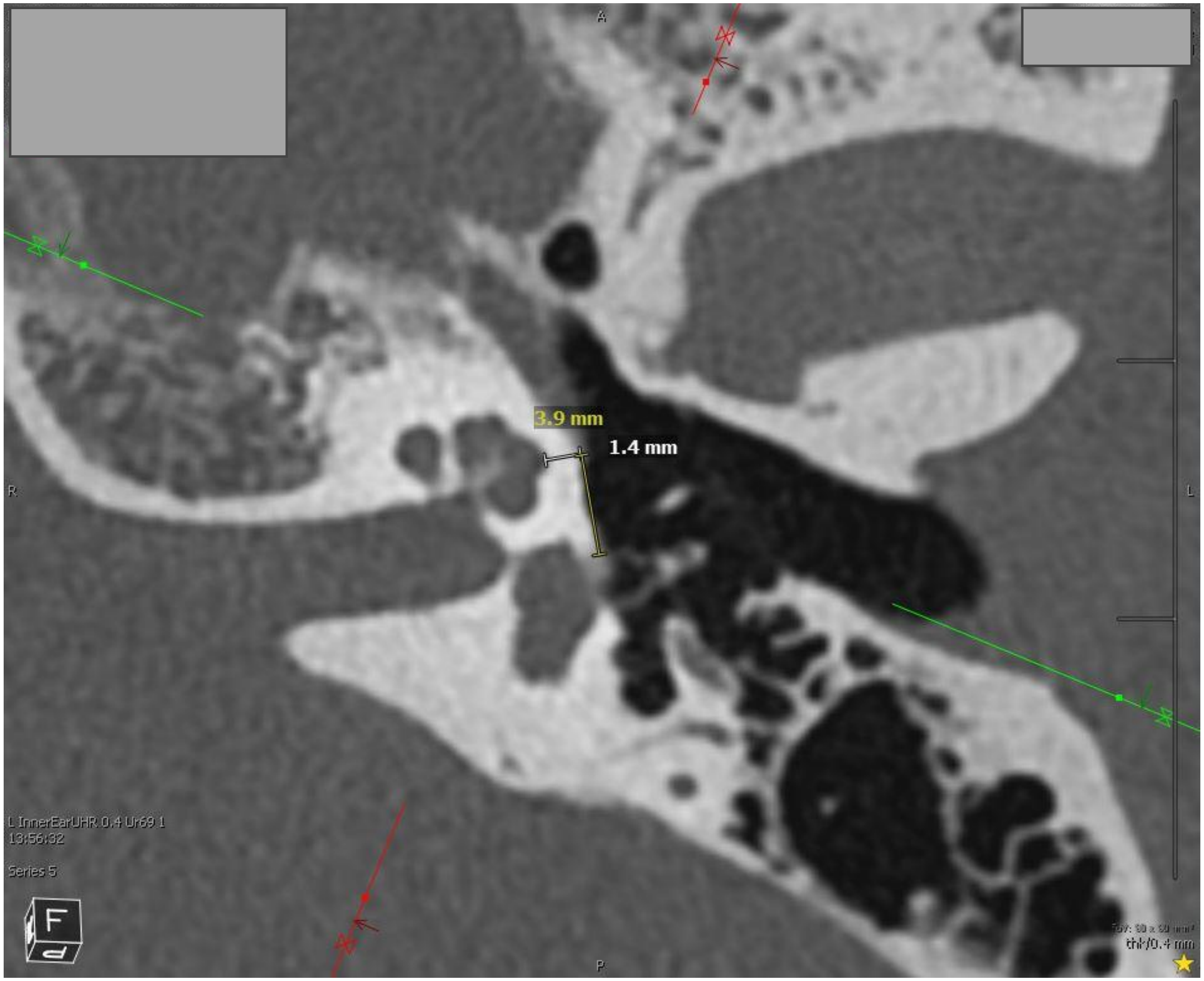

<u>Oval Window Distance (AP)</u>

Axial MPR in the standard plane was utilized to assess the AP distance from the anterior oval membrane to the cochlear promontory found 90º from the lateral membranous CA.

Step 1: Identify anterior border of oval window membrane and initiate AP measurement

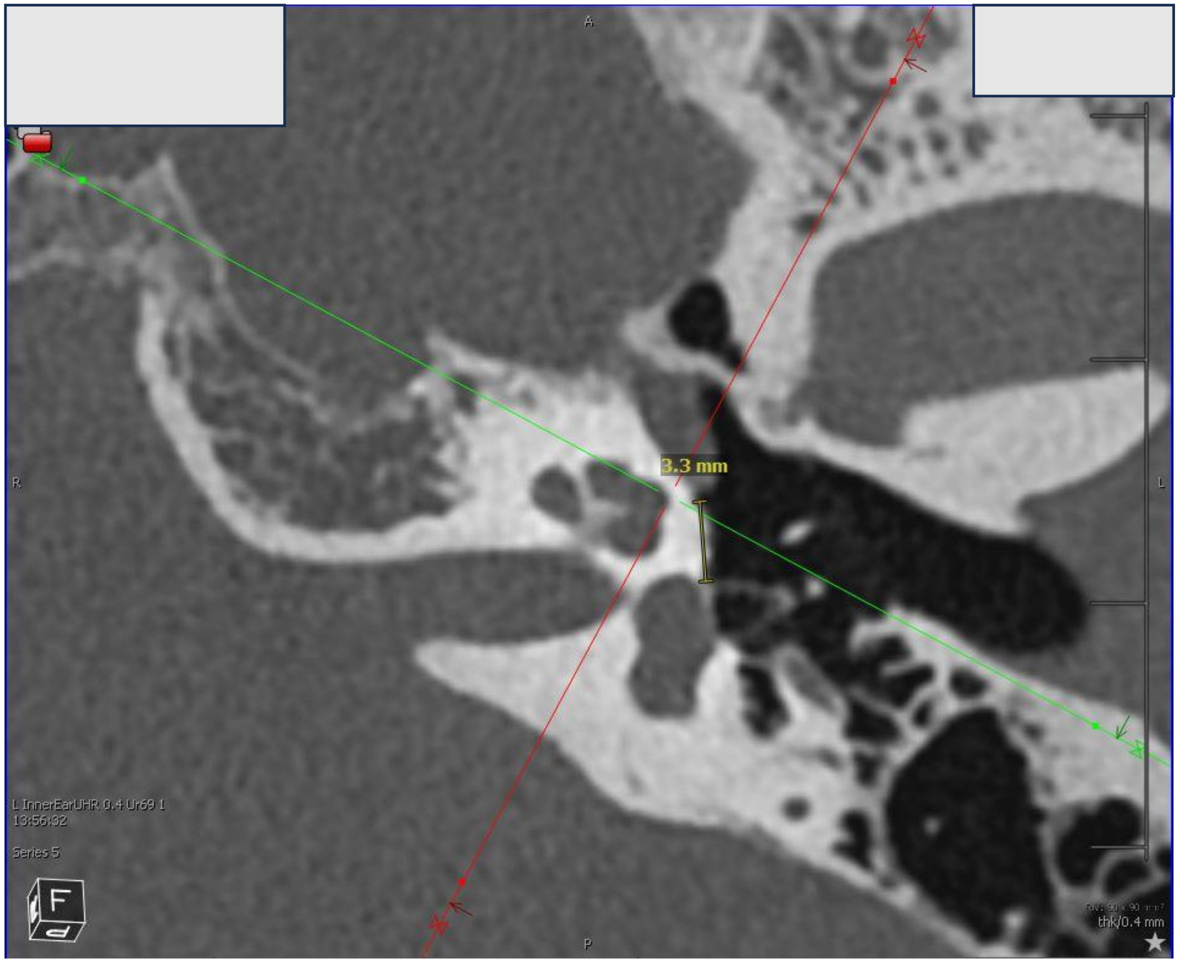

Step 2: Scroll through axial images until reach level of lateral apex to complete measurement

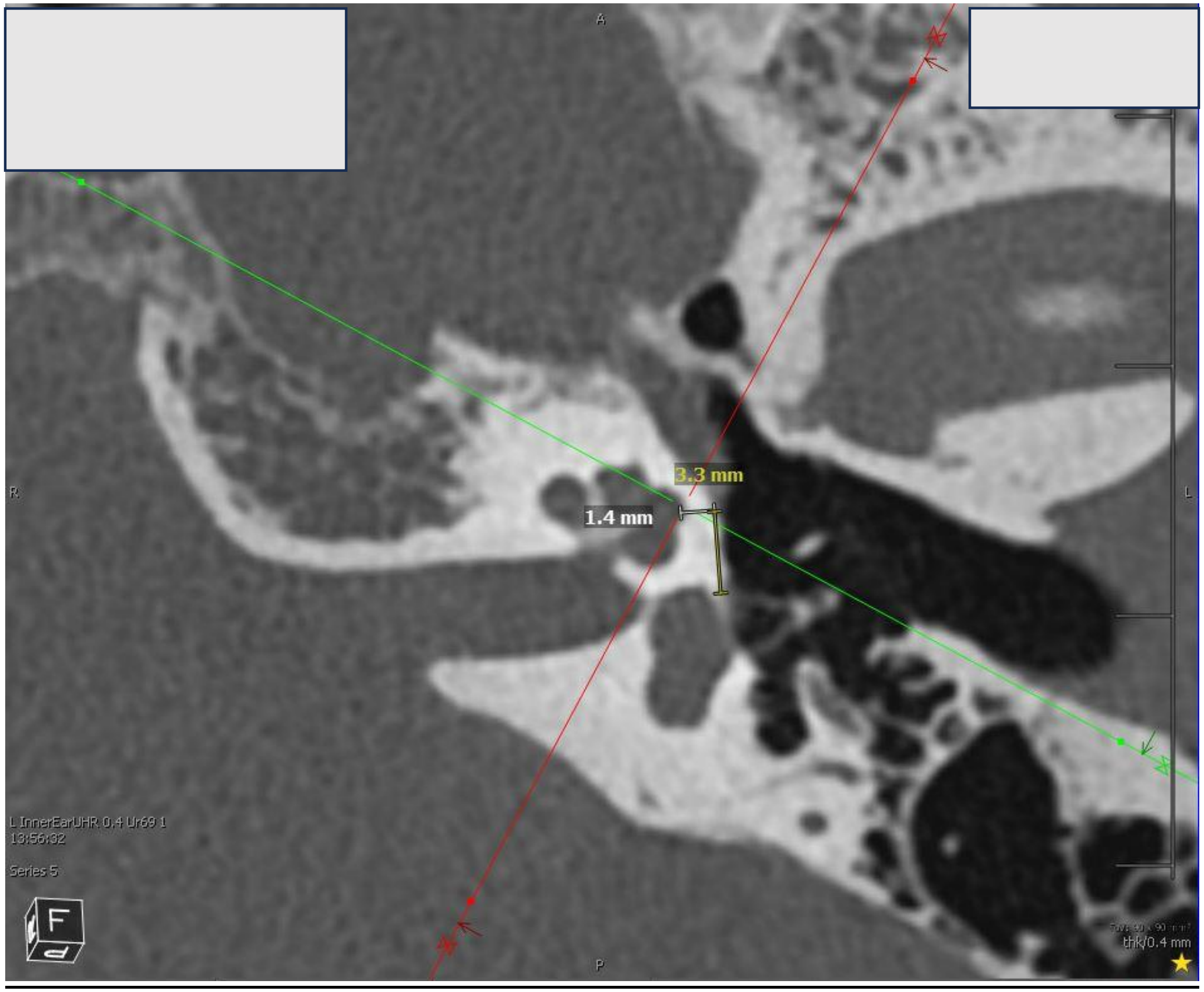

<u>Cochleariform Process Distance (AP + Height)</u>

Axial MPR in the standard plane was utilized to assess the AP distance from the inferior cochleariform process to the cochlear promontory found 90º from the lateral membranous CA. Sagittal MPR in the standard plane was utilized to assess the height of the inferior cochleariform process from the lateral aspect of the membranous CA.

AP Step 1: Identify inferior border of cochleariform process and initiate AP measurement

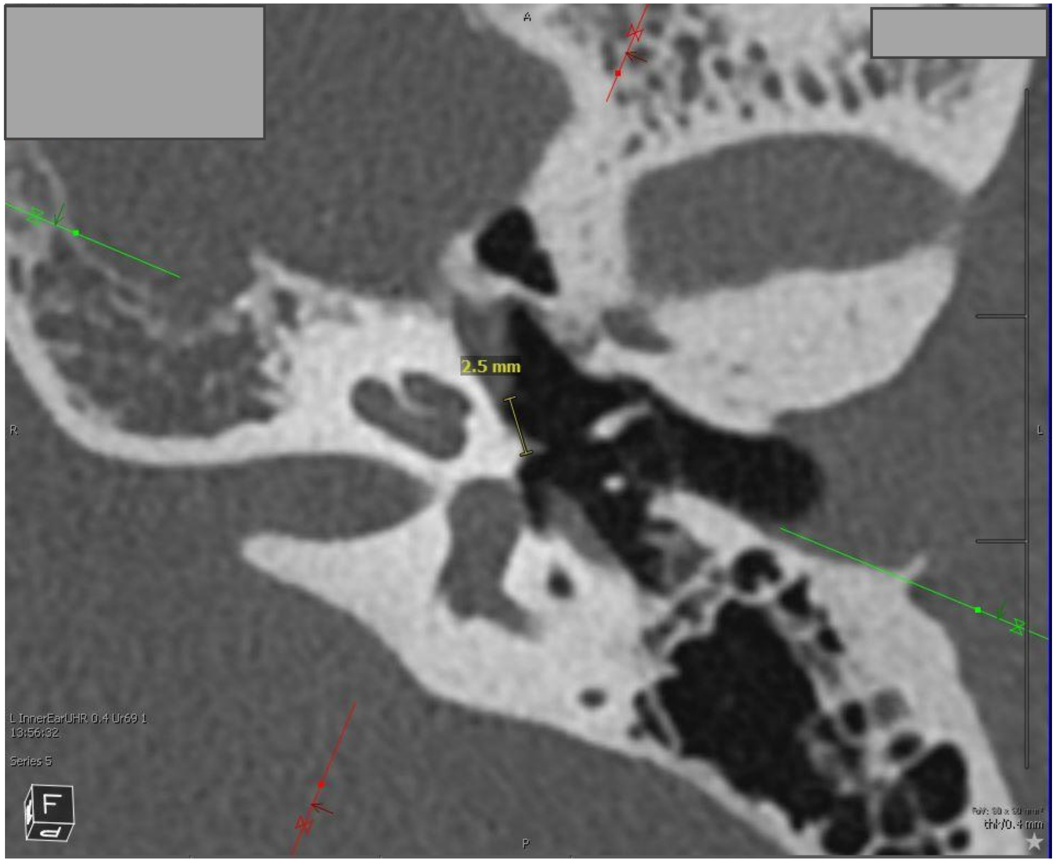

AP Step 2: Scroll through axial images until reach level of lateral apex to complete measurement

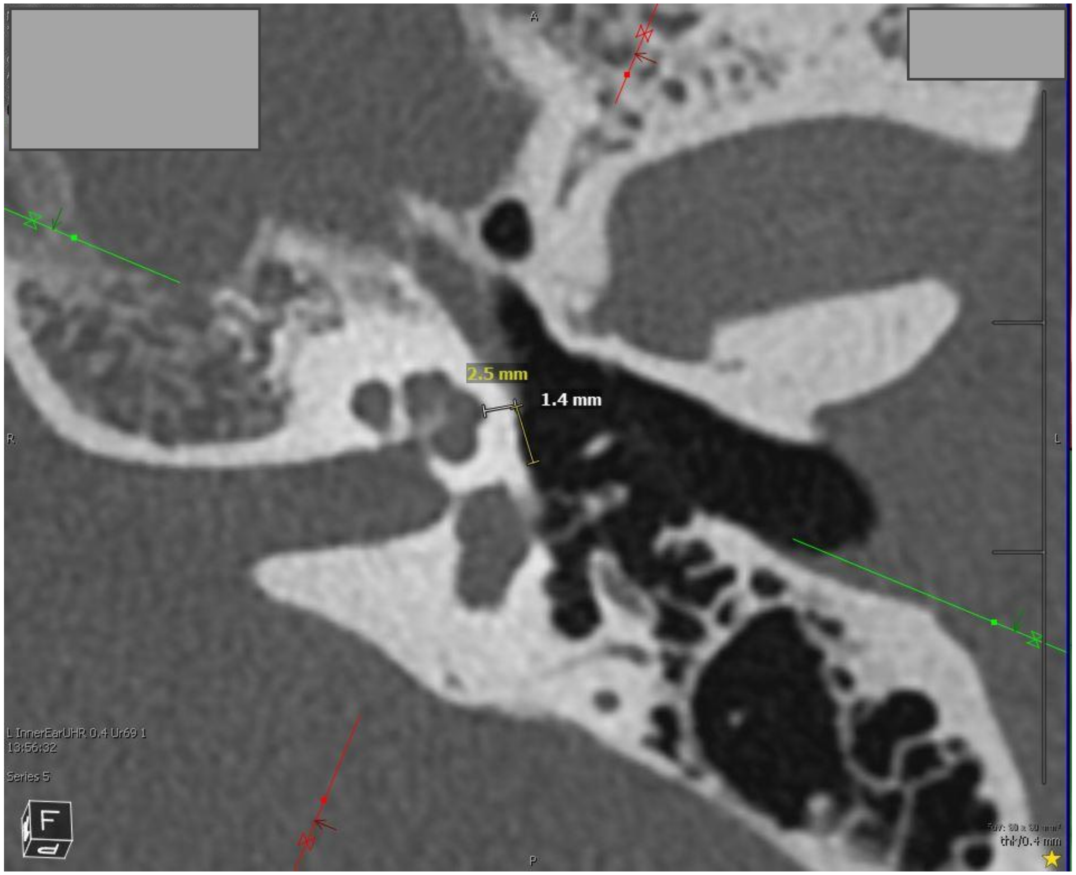

Height Step 1: Identify level of lateral border of cochlear apex on sagittal images

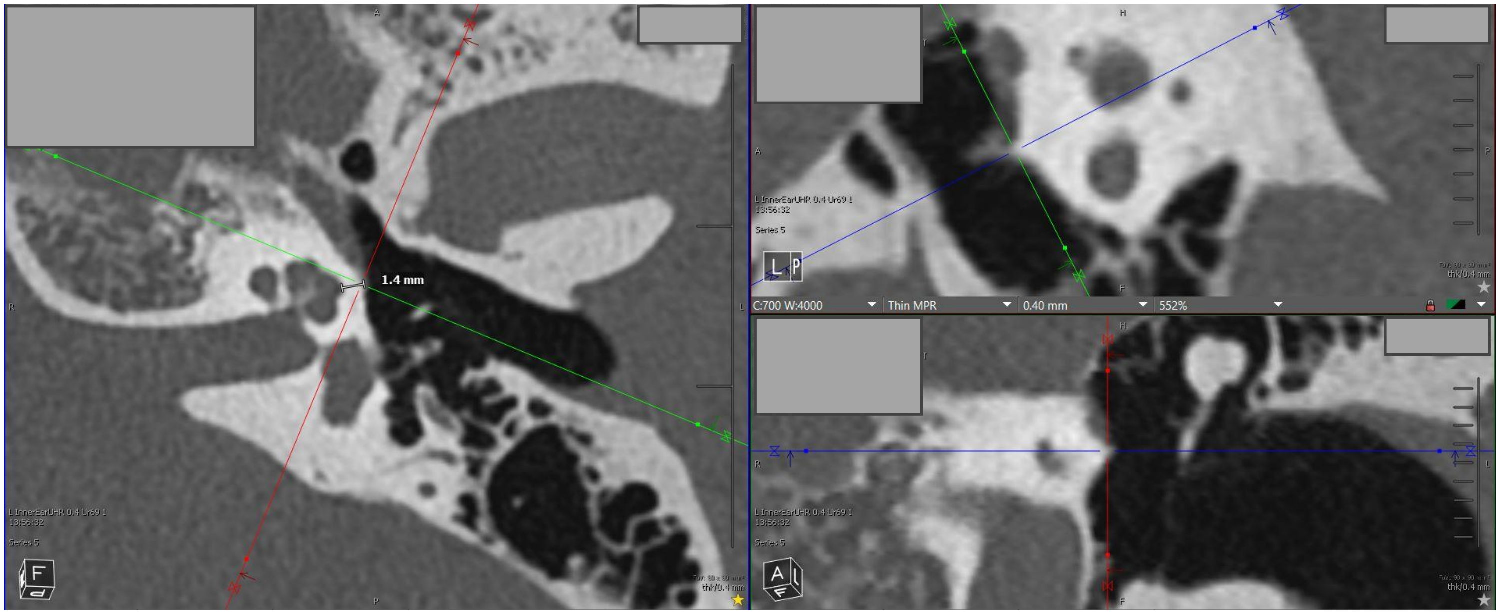

Height Step 2: Scroll through sagittal images until identify inferior border of cochleariform process and complete height measurement

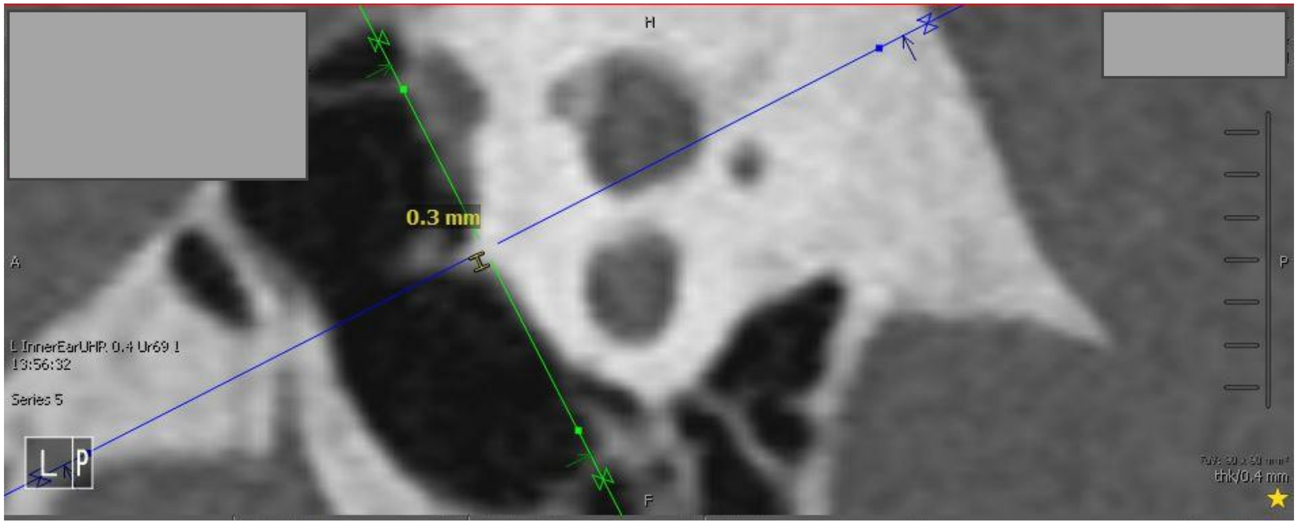

<u>Tensor Tympani Level</u>

Sagittal MPR in the standard plane was utilized to assess the height of the tensor tympani (inferior border) from the lateral aspect of the membranous CA. The inferior border of the tensor tympani was chosen, as this was felt to be more easily recognizable and relevant surgically compared to the muscle belly midpoint.

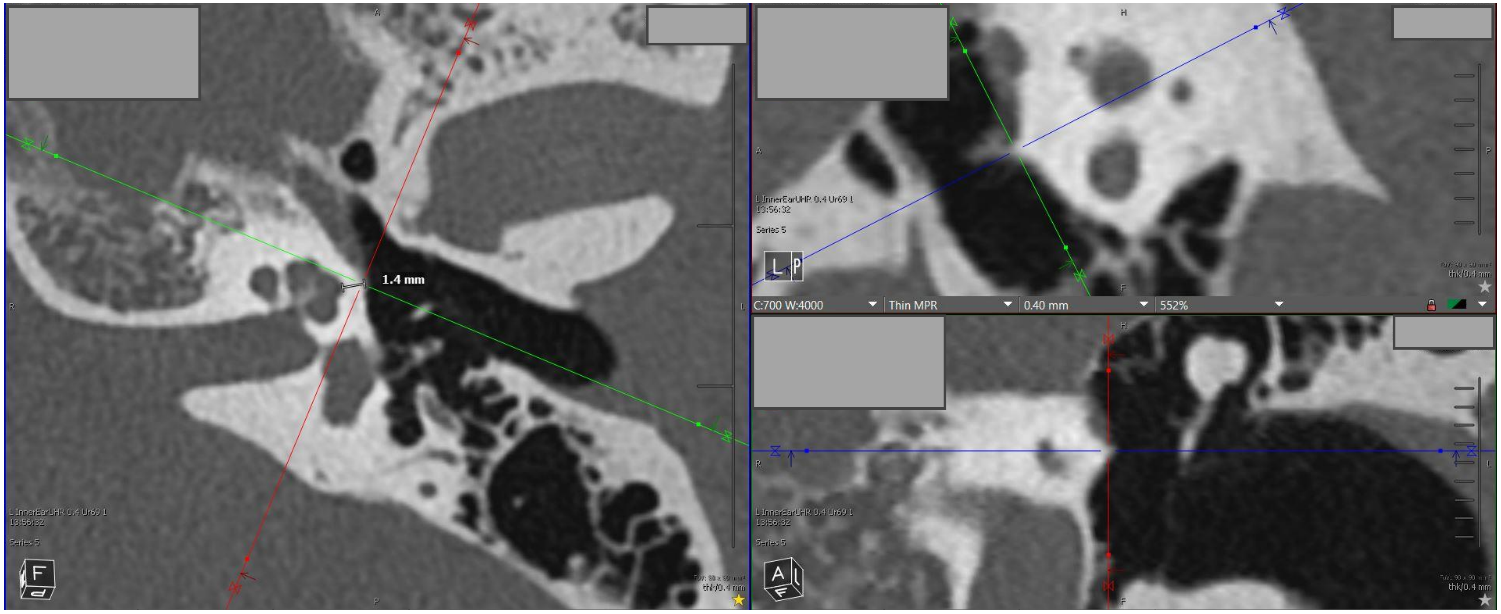

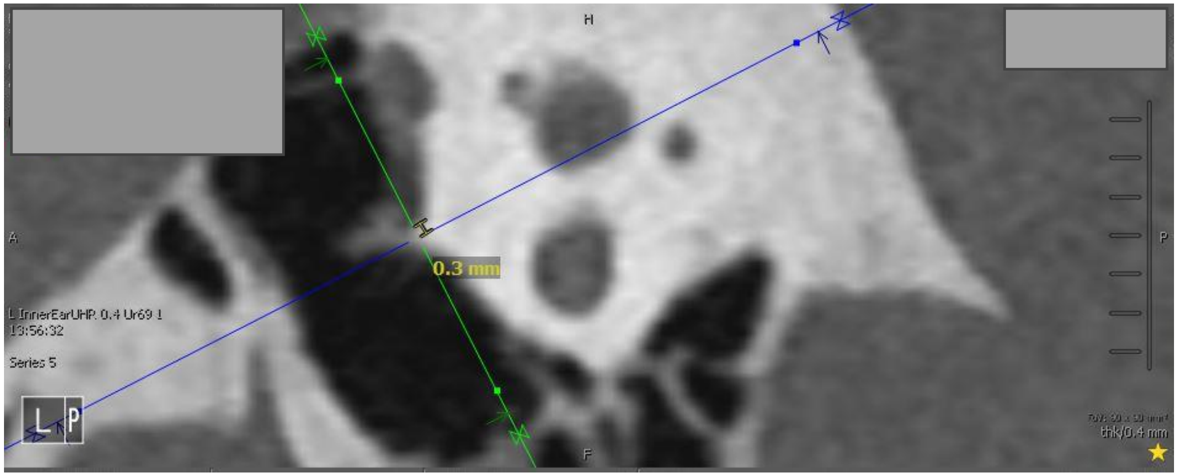

Labyrinthine Facial Nerve Distance

Coronal MPR in the standard plane was utilized to measure the shortest distance from the labyrinthine facial nerve to the most apical 360° of the cochlear apex.

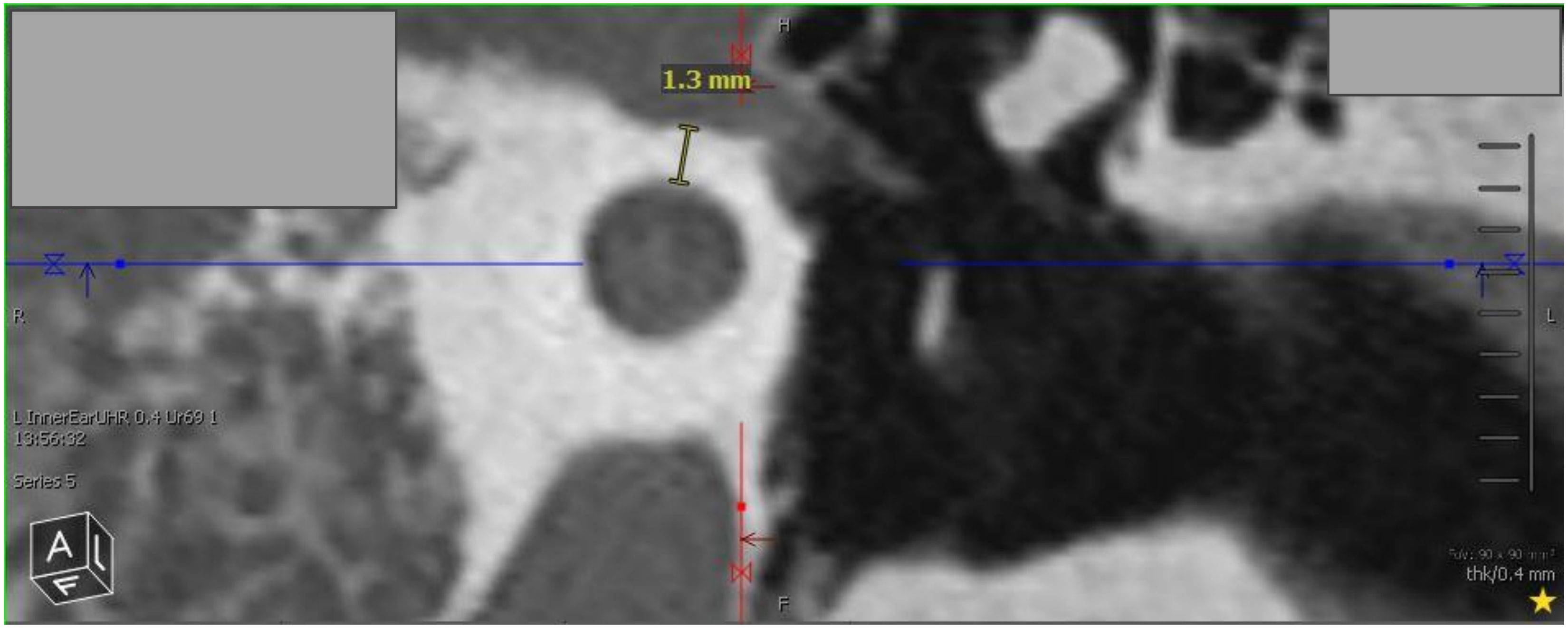

Carotid Distance

Coronal MPR in the standard plane was utilized to measure the shortest distance from the internal carotid artery to the most apical 360° of the cochlear apex. If the internal carotid artery was not visualized on coronal cut, it was labelled as *NA*.

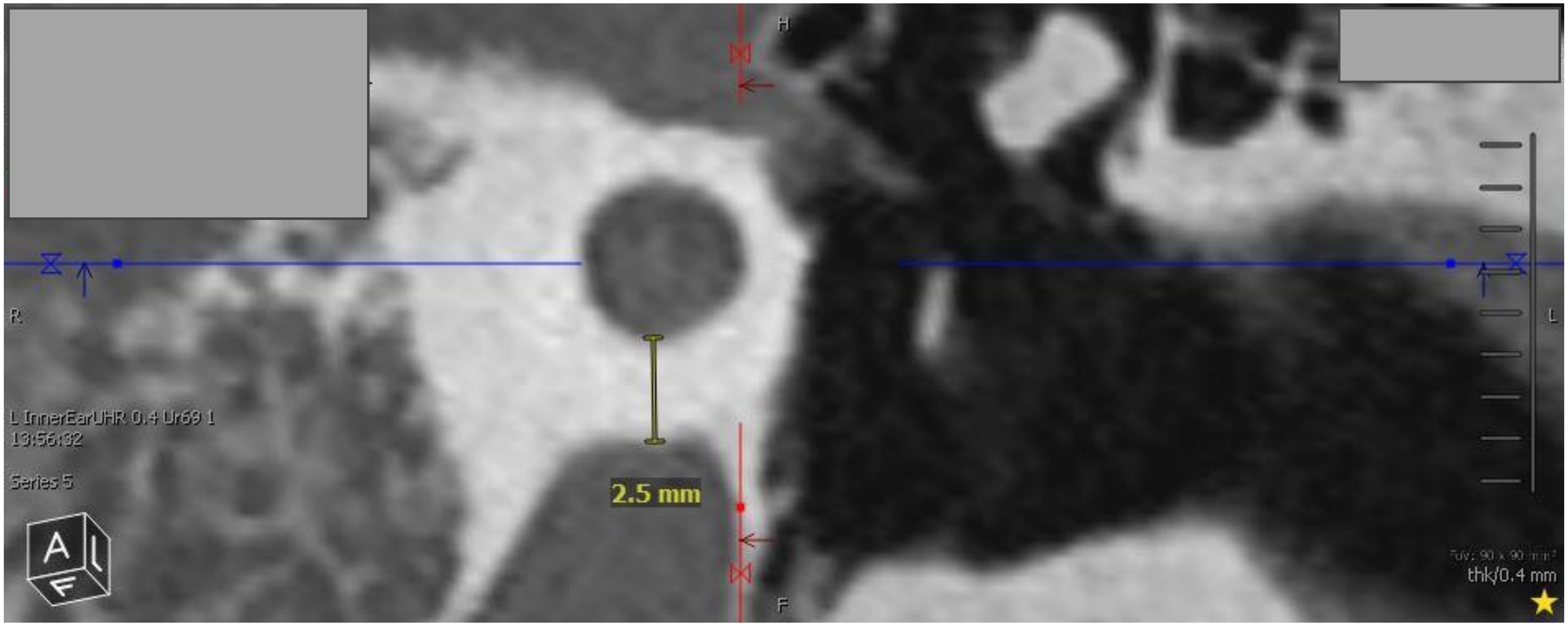

Special Measures

These measures were not completed utilizing the standard MPR plane.

Anterior Carotid Relationship

Axial MPR was aligned with the horizontal segment of the internal carotid artery (ICA). A vector was drawn parallel to the anterior border of the horizontal segment of the ICA.

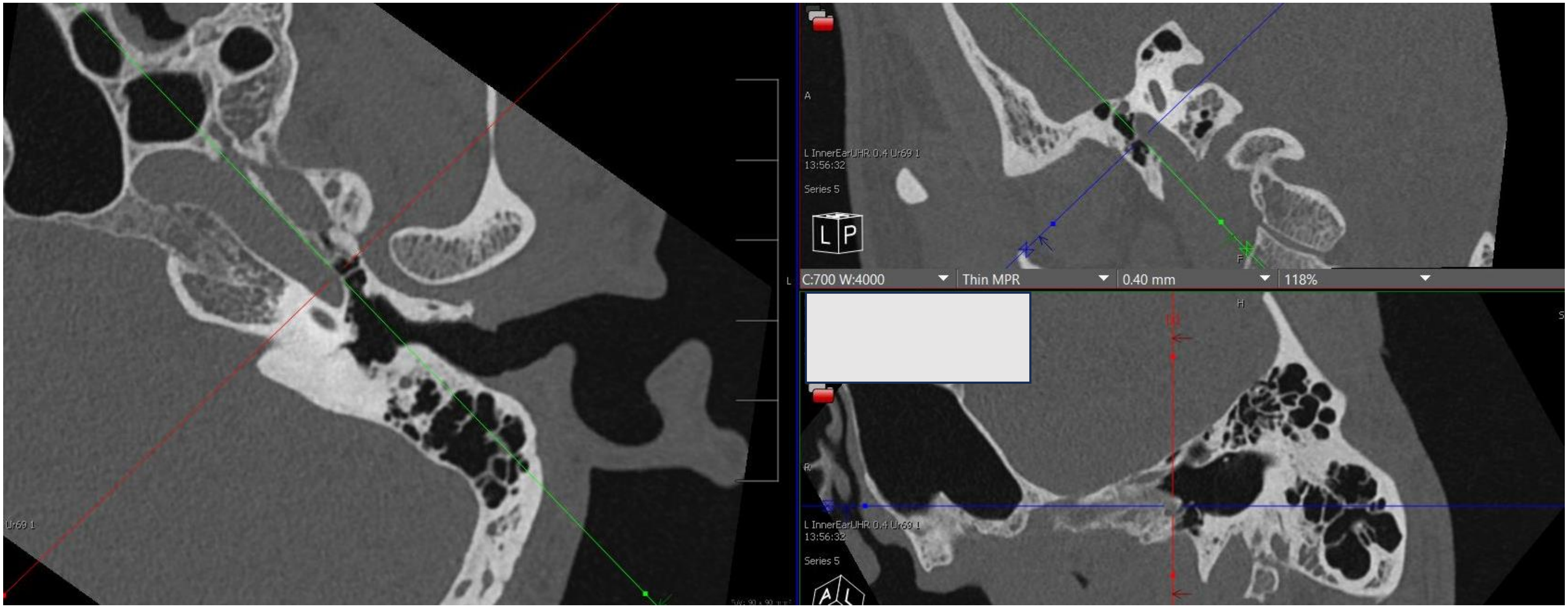

The distance between the vector and cochlear apex was then measured in axial plane.

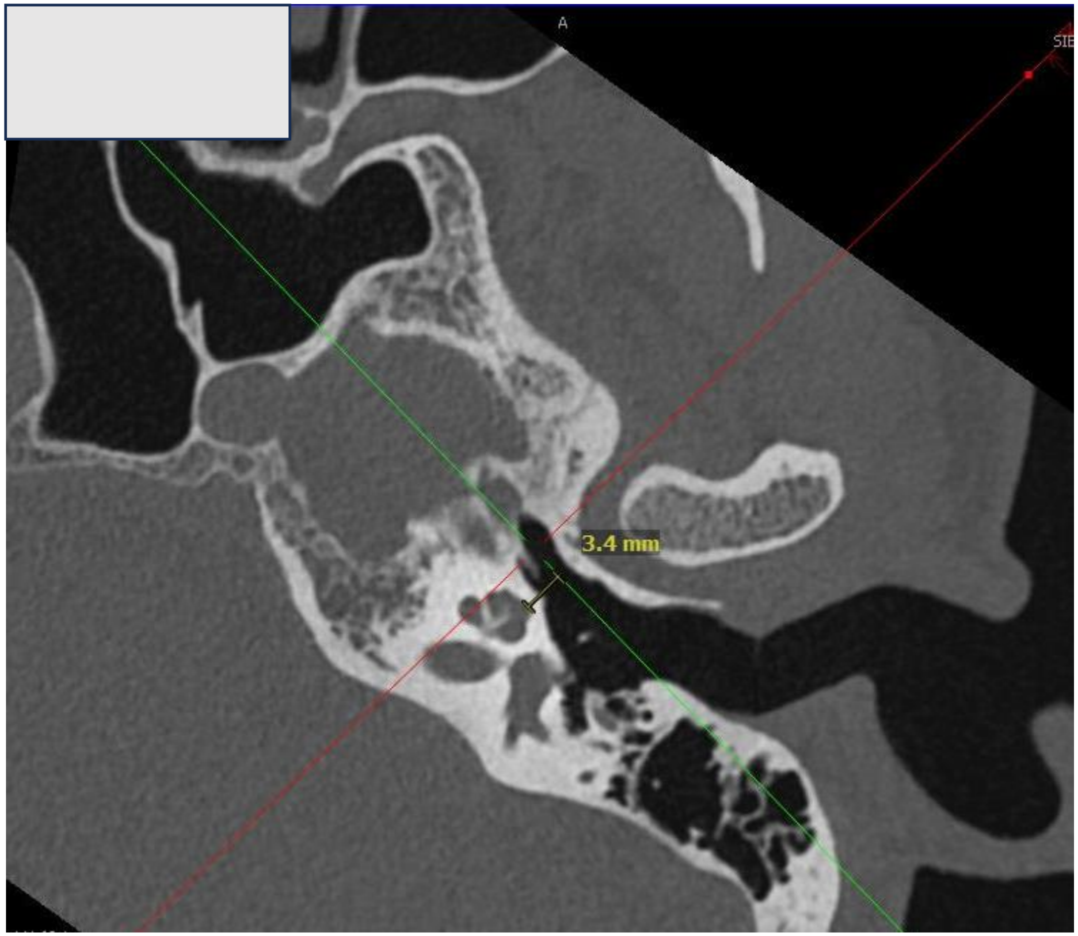

Stapes Vector (From Stapes and Round Window)

Axial and sagittal MPR were aligned with the stapes footplate, allowing the coronal MPR to bisect the anterior and posterior crura of the stapes. A vector was drawn through the anterior and posterior crura of the stapes.

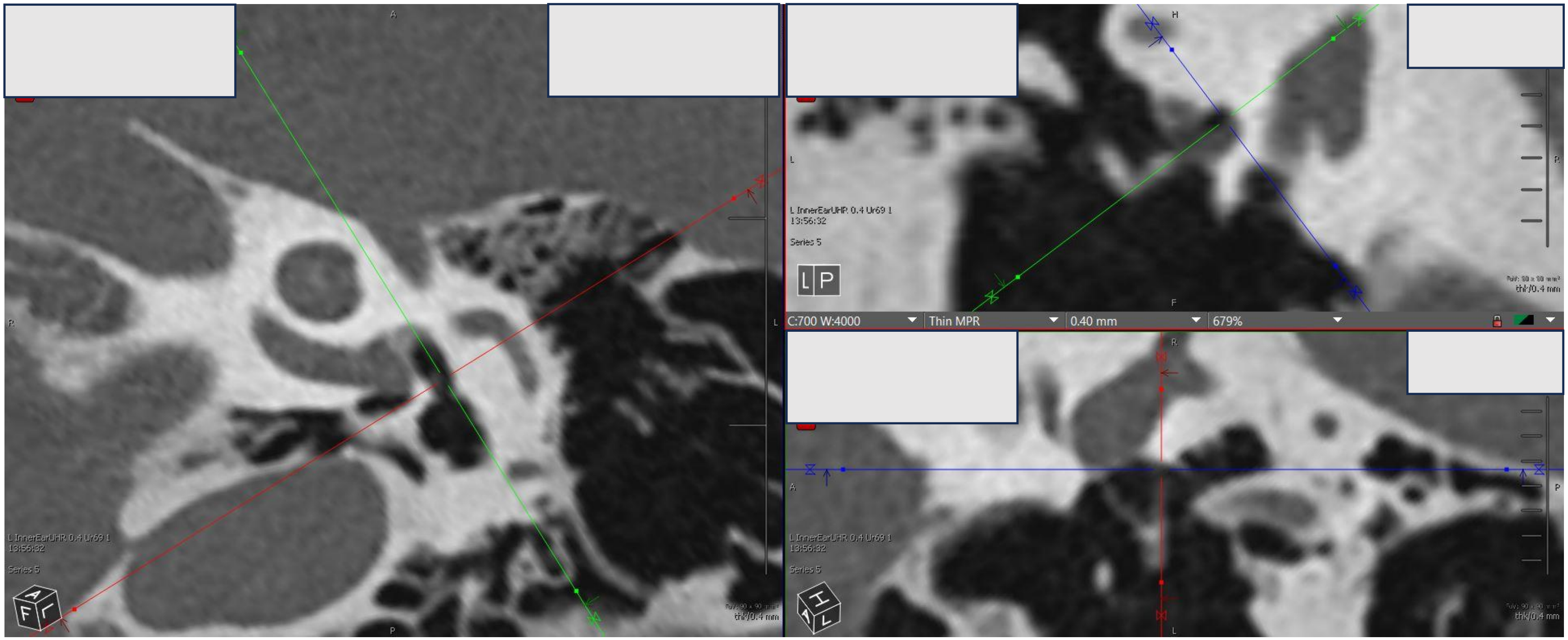

The vector (V1) was followed anterior on sagittal imaging until the cochlear apex was identified, and distance between the superior CA and vector was measured.

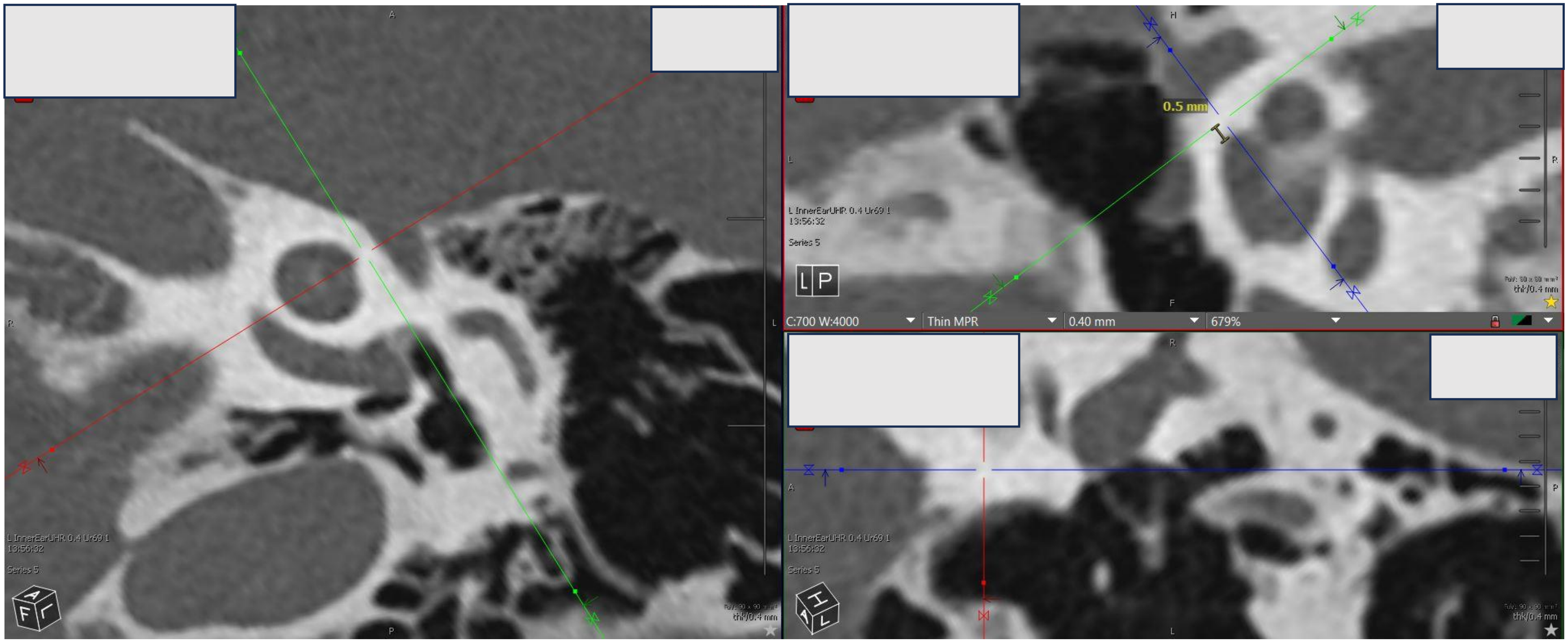

A new vector (V2) was measured parallel to V1 at the lateral anterior aspect of the round window.

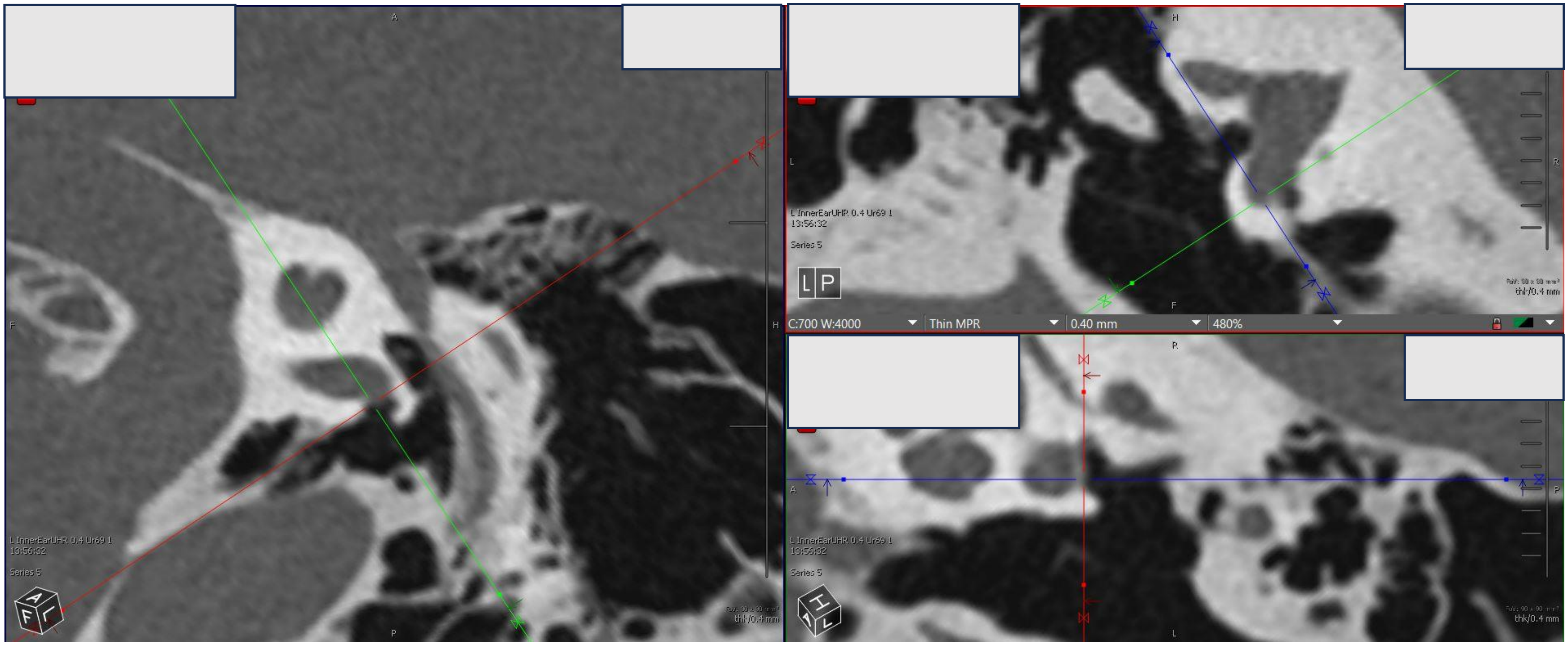

V2 was following anteriorly on sagittal image, and the distance between the inferior CA and vector was measured.

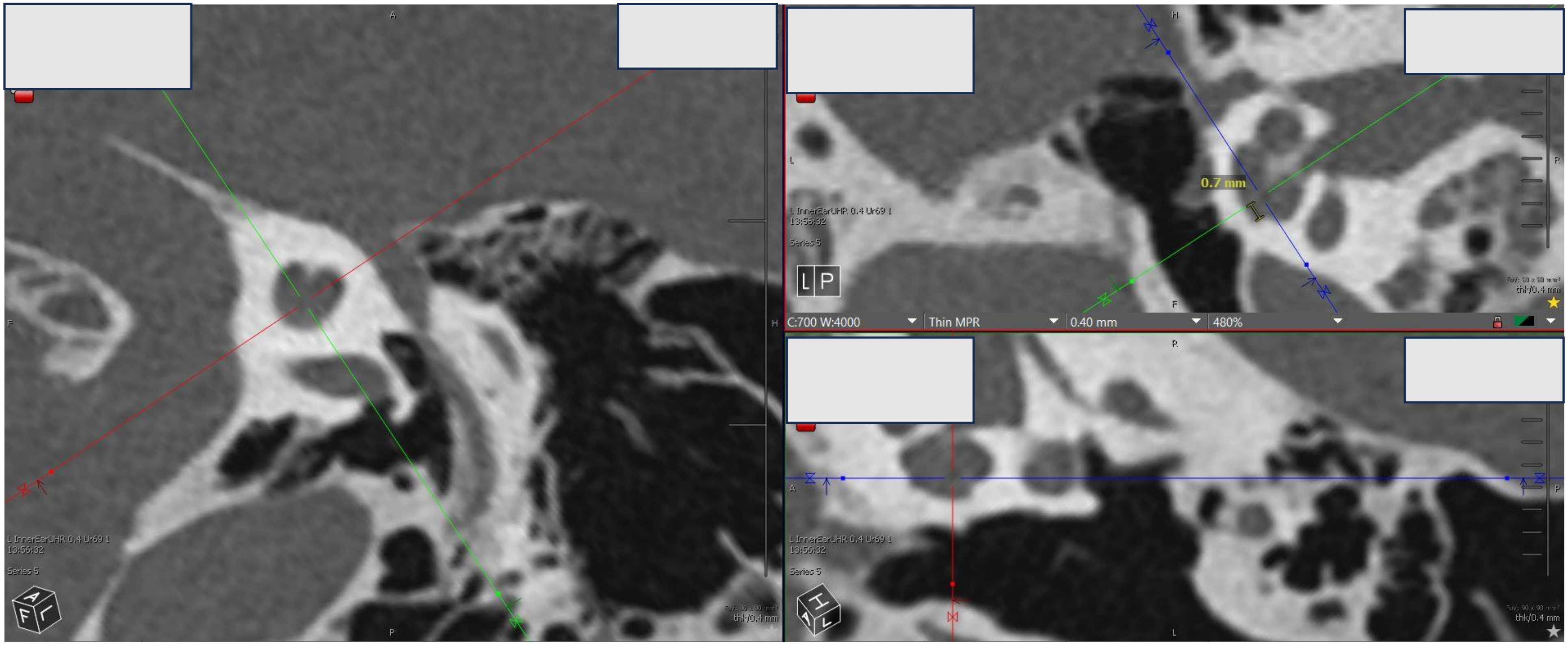

Supplement: Supplementary file 1 [file on9-4-e060-s001.pdf]
